# Plasmonic Molecular Entrapment for Label‐Free Methylated DNA Detection and Machine‐Learning Assisted Quantification

**DOI:** 10.1002/advs.202503257

**Published:** 2025-05-08

**Authors:** Muhammad Shalahuddin Al Ja'farawy, Vo Thi Nhat Linh, Chaewon Mun, Jun‐Yeong Yang, Jun Young Kim, Rowoon Park, Sung‐Gyu Park, Dong‐Ho Kim, Min‐Young Lee, Ho Sang Jung

**Affiliations:** ^1^ Advanced Bio and Healthcare Materials Research Division Korea Institute of Materials Science (KIMS) Changwon Gyeongnam 51508 South Korea; ^2^ Advanced Materials Engineering Korea National University of Science and Technology (UST) Daejeon 34113 South Korea; ^3^ School of Convergence Science and Technology Medical Science and Engineering Pohang University of Science and Technology (POSTECH) Pohang Kyungbuk 37673 South Korea

**Keywords:** DNA methylation, hotspot engineering, label‐free diagnosis, plasmonic materials, surface‐enhanced Raman scattering

## Abstract

Epigenetic DNA methylations are linked to the activation of oncogenes and inactivation of tumor suppressor genes. A reliable and label‐free method to quantitatively measure DNA methylation levels is essential for diagnosing and monitoring methylation‐related diseases. Herein, plasmonic molecular entrapment (PME) method assisted SERS as facile strategy for trapping and label‐free sensing of DNA methylation, utilizing in situ surface growth of plasmonic particle in the presence of target analytes, are developed. This highly sensitive and adaptable technique forms hotspot sites around target analytes, overcoming mismatch geometrical properties and producing a strong electromagnetic field that leads to significant SERS signal enhancement. The PME method effectively profiles and quantifies DNA methylation, demonstrating robust capabilities for DNA analysis. A logistic regression (LR)‐based machine learning accurately quantifies and classifies methylation levels in clinical serum samples of colorectal cancer and normal patients with high sensitivity, specificity, and accuracy, highlighting the feasibility of this technique. The developed PME method combined with machine learning offers promising sensing techniques for disease screening and diagnosis, marking a significant advancement in disease detection and patient care.

## Graphical abstract

Plasmonic molecular entrapment (PME) is label‐free detection and quantification method for DNA methylation. This technique provides direct hotspot site generation around target analytes, overcoming mismatch geometrical properties. The PME demonstrates superior SERS performance and quantification capability, offering promising potential for epigenetic disease detection. Machine learning analysis successfully quantifies and classifies DNA methylation levels in human serum, highlighting its diagnostic prospects.

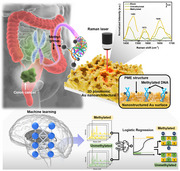

## 1 Introduction

Genetic and epigenetic modifications are hallmarks of cancer.^[^ ^1^ ^]^ DNA methylation, the most common epigenetic modification in the mammalian genome, plays a crucial role in the activation of oncogenes.^[^ ^1^, ^2^, ^3^ ^]^ Aberrant DNA methylation can silence tumor suppressor genes, leading to carcinogenesis and tumor progression.^[^ ^3^, ^4^, ^5^ ^]^ In addition, the methylation of tumor promoter genes also provides a valuable cancer‐specific information and insights into tumor progression, making it promising as an epigenetic biomarker.^[^ ^4^, ^6^ ^]^ Several techniques have been explored for DNA methylation analysis, including methylation‐sensitive restriction enzymes (MSREs), sodium bisulfite conversion, and affinity purification.^[^ ^7^, ^8^, ^9^ ^]^ The sodium bisulfite conversion, the most commonly used method, converts unmodified cytosine into uracil while leaving methylated cytosine unchanged, offering excellent quantification capabilities and cost‐effectiveness.^[^ ^9^, ^10^ ^]^ However, harsh process, time consuming, and poor detection sensitivity become its limitation.^[^ ^9^, ^11^ ^]^ Recently, alternative methods, such as electrochemical, electric, and optical biosensor, have been extensively developed.^[^ ^12^, ^13^, ^14^, ^15^, ^16^ ^]^ Despite their advancement, most of these techniques remain complex and often face challenges in accurately quantifying the degree and ratio of methylation, which are critical for assessing cancer progression.^[^ ^17^ ^]^ Given these challenges, developing new sensing technologies with high accuracy, speed, affordability, and capability to identify and quantify DNA methylation is required.

Over the past decades, surface enhanced Raman scattering (SERS) has established as a powerful technique known for its high sensitivity, simplicity, and highly multiplexed analysis, enabling direct label‐free quantification of biological samples.^[^ ^18^, ^19^, ^20^ ^]^ In principle, SERS is molecular vibrational sensing method that amplifies the inelastic light scattering of molecules by several orders of magnitude due to electromagnetic field generation on plasmonic nanostructure.^[^ ^18^, ^20^, ^21^, ^22^ ^]^ In label‐free SERS sensing, subtle spectral variations induced by single‐point mutations, methylation changes, or even the secondary structures, can be reliably differentiated by leveraging the intense local electromagnetic field to amplify the intrinsically weak Raman scattering, making it promising for DNA methylation quantification.^[^ ^17^, ^23^ ^]^ Colloidal plasmonic nanoparticle in aggregated or dimer nanostructure form often represents an attractive strategy to investigate DNA molecule by positioning them into plasmonic gap or hotspot, where the confined intense electromagnetic field can significantly contribute to SERS enhancement.^[^ ^17^, ^24^, ^25^, ^26^ ^]^ However, irreproducible and irregular particle aggregation may occur in multicomponent sample, which can notably affect the reproducibility and uniformity of SERS signal generation.^[^ ^27^ ^]^ Plasmonic nanostructures precisely formed on solid substrates offer a promising biosensing platform due to their uniform particle distribution, which can potentially enhance the reproducibility and reliability of SERS signal generation.^[^ ^23^, ^28^, ^29^ ^]^ Nevertheless, the low signal‐to‐noise ratio (SNR) due to the random distribution of analytes on the substrates, along with the difficulty in positioning DNA on the hotspot sites, remains a challenge.^[^ ^29^ ^]^ As an alternative, supported techniques such as electrochemical method or chemical ligand‐functionalization are required to localize molecules into hotspot regions.^[^ ^30^, ^31^, ^32^ ^]^ However, these methods suffer from high limit of detection (LOD) due to mismatched geometrical properties of the target and hotspot site, lengthy pretreatment process and complex binding dependency, remaining significant drawbacks.^[^ ^29^, ^30^, ^33^ ^]^ Therefore, an innovative and feasible SERS approach with high sensitivity is crucial for the precise quantification of DNA methylation.

**Figure 1:**
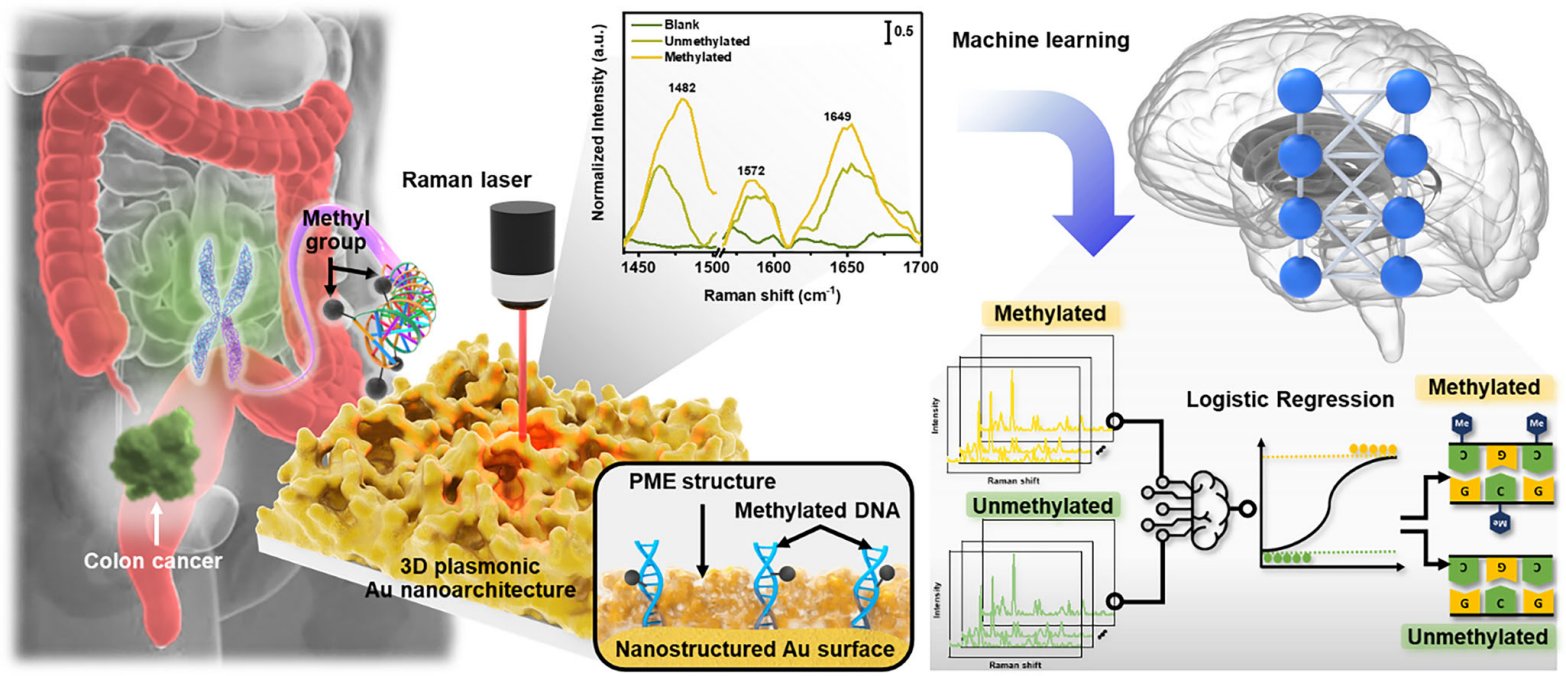
Schematic illustration of the PME method for label‐free DNA methylation detection and quantification.

To address the aforementioned concerns, we developed a plasmonic molecular entrapment (PME) method‐assisted SERS as a facile strategy for trapping and label‐free sensing of DNA methylation, as illustrated in **Figure** **1**. PME enables precise molecular localization and entrapment through in situ surface growth of Au particles, highlighting hotspot formation around target analytes and ensuring their direct positioning within plasmonic hotspots rather than relying on random adsorption. This targeted approach significantly enhances label‐free SERS sensing and improves sensitivity. This method leverages the surface‐directed reduction properties of hydroxylamine hydrochloride (HH), which selectively reduces Au ions in the presence of nucleation sites.^[^ ^34^, ^35^, ^36^ ^]^ By utilizing this characteristic, PME achieves molecular entrapment via the in situ growth of Au particles on the nucleation surface. To support this process, a porous Au nanosponge (AuS) with a mesoporous structure was employed as a 3D nucleation and adsorption site, promoting Au reduction and DNA adsorption. This configuration provides a strong and uniform electromagnetic field distribution, enabling effective DNA adsorption and localization. The results demonstrated not only superior Raman signal enhancement, but also significant morphological evolution, overcoming limitations associated with mismatched geometrical properties and enabling a highly sensitive, label‐free detection technique. This approach was further investigated for DNA methylation quantification through SERS intensity variations at various methylation levels (0–4) and ratios (9.5%–18.2%), demonstrating the capability of this technique for DNA analysis. Furthermore, the feasibility of PME for disease diagnosis was demonstrated using clinical serum samples of colorectal cancer and normal patients. Logistic regression (LR)‐based machine learning model was applied to classify and quantify global methylation levels based on Raman spectra variations, achieving remarkable results with overall sensitivity, specificity, and accuracy of 100%, 98.3%, and 99.2%. The results demonstrated competitive performance relative to conventional methods (Table S1, Supporting Information), while offering a rapid, facile, label‐free and low cost. Therefore, PME offers a facile strategy in label‐free SERS sensing, demonstrating a promising potential for accurate detection of a wide range of target analytes with high precision and adaptability.

## 2 Result and Discussion

### 2.1 Developing PME

**Figure 2:**
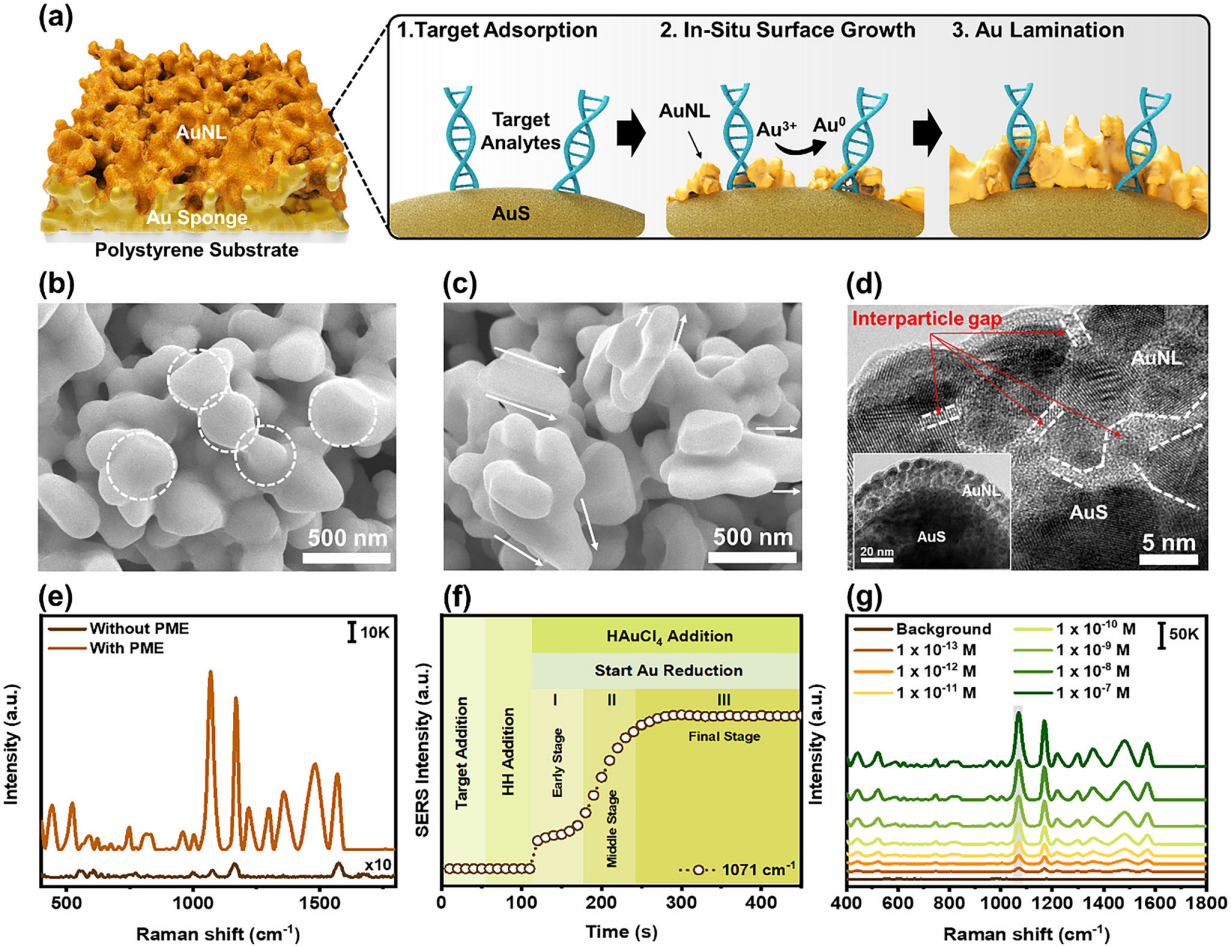
Development of PME method. a) Schematic illustration of the PME method process, SEM images of AuS after PME b) without target analyte and c) with target analyte, d) HR‐TEM and TEM (inset) images of the laminated area on AuS surface in the presence of target analyte, e) SERS enhancement in PME development (scale bar refers to SERS intensity), f) Real‐time monitoring of SERS signal changes at 1 071 cm^−1^ during PME process, g) SERS performance of PME method at various 4‐ATP concentration (scale bar refers to SERS intensity).

The PME method was developed using an Au nanostructure substrate as a nucleation site, with the addition of Au precursor and HH as trapping agents, in the presence of target analytes (**Figure** **2**a). A porous Au nanosponge (AuS) with a spherical particle‐like polyhedrons (SPLPs) structure on the polystyrene substrate was selected as the nucleation site due to its simplicity, high surface area, and efficient adsorption capability (Figure S1a, Supporting Information). This nanostructure provides a 3D nucleation site with mesoporous size of 43.3 ± 8.2 nm, offering volumetric field beneficial for target analytes adsorption in both liquid‐ and solid‐state measurements.^[^ ^37^ ^]^ Furthermore, AuS consists of a fundamental face‐centered cubic (FCC) crystal structure, predominantly composed of (111) and (200) facet (Figure S1b,c, Supporting Information), enabling the effective PME investigation due to the preferential adsorption of some target analytes on these planes.^[^ ^38^ ^]^ According to previous studies, HH functions as a growth medium that reduces Au ions only when a nucleation site is present, due to its autocatalytic surface growth properties.^[^ ^34^, ^35^, ^36^, ^39^ ^]^ Building on this, we speculated that the reduction of Au ions in the presence of both nucleation site and target analyte would promote molecular entrapment on the nucleation surface through in situ surface growth of Au nanolaminates (AuNL). Thus, PME was developed through the sequential addition of target analytes, HH, and Au precursor onto the AuS substrate. The Raman reporter 4‐aminothiophenol (4‐ATP) was used as target analyte for investigating PME method due to its preference for adsorption on the (111) facet, as reported elsewhere.^[^ ^38^ ^]^ This preferential adsorption enables the recognition of trace target analytes in PME through morphological changes. The scanning electron microscope (SEM) images clearly show the morphological change of AuS after PME in the absence (Figure 2b; Figure S2a, Supporting Information) and presence (Figure 2c; Figure S2b, Supporting Information) of 4‐ATP. In the absence of 4‐ATP, AuNL non‐selectively grows on the AuS, maintaining its initial quasi‐spherical shape with round edges and displaying a broad surface plasmon resonance (SPR) peak from 500 to 750 nm (Figure S2c, Supporting Information). Notably, in the presence of 4‐ATP, the AuNL exhibited growth primarily in the transverse direction, resulting in the formation of protruded AuS. This morphological evolution led to a broad SPR peak, significantly amplified at 696 nm, attributed to the formation of these protrusions.^[^ ^40^, ^41^ ^]^ To investigate the growth mechanism of AuNL in the absence and presence of target analytes, the high‐resolution transmission electron microscope (HR‐TEM) was performed on the boundary regions of the AuNL. In the absence of target analyte, the AuNL particles appeared connected and coalesced onto the primary particles of AuS (Figure S3a, Supporting Information). Interestingly, in the presence of target analyte, the interparticle gaps of 1.14 ± 0.36 nm were observed within the AuNL region (Figure 2d; Figure S3b, Supporting Information). This observation suggests that the adsorbed target analyte on the Au surface could block the deposition of newly formed Au atoms on particular facet, as reported elsewhere.^[^ ^42^, ^43^, ^44^ ^]^ To further investigate the impact of interparticle gap formation, we performed Raman analysis of 4‐ATP on both pristine AuS and PME (Figure 2e). Without PME, weak Raman signals of 4‐ATP were observed at 1 071, 1 171, and 1 569 cm^−1^ (Table S2, Supporting Information), due to the surface selection rule that favors vibrational modes with significant polarizability.^[^ ^45^, ^46^ ^]^ Interestingly, several undetected peaks at 442, 1 003, 1 217, 1 301, 1 358, and 1482 cm^−1^ underwent a substantial enhancement of ≈200‐fold in PME. This result suggests that the formation of interparticle gaps around target analytes enhances electromagnetic field generation, amplifying all vibrational modes regardless of their orientation, thereby making weak or undetectable Raman peaks visible.

To determine the mechanism of PME method for Raman signal enhancement, in situ real‐time monitoring of 4‐ATP signal at 1 071 cm^−1^ was observed on the Raman spectroscopy (Figure 2f). The Raman signal of 4‐ATP was significantly enhanced after the Au reduction began at t = 120 s. The amplification trend progressed through three phases: I) early stage (t = 120–170 s), II) middle stage (t = 170–280 s), and III) final stage (t > 280 s). Based on the SEM image at different reaction times (Figure S4, Supporting Information), protrusion growth was observed, increasing over time, consistent with in situ Raman signal monitoring. At early stage, small protrusions were started growing, which explains the gradual amplification of the Raman signal. At middle stage, larger and sharper protrusions were observed explaining the significant Raman enhancement due to hotspots generation and edge effect. At final stage, similar morphologies were observed, indicating that the reaction had completed, resulting in the saturation of the Raman signal. We speculated that the molecular entrapment on the nucleation surface was the primary factor contributing to the enhancement of the Raman signal. To confirm this, we examined various conditions, including AuS, AuS + HH, AuS + HAuCl_4_, and AuS + HH + HAuCl_4_ (Figure S5a,b, Supporting Information). Significant Raman signal enhancement of 4‐ATP was observed only after adding HH and Au precursor to AuS. No further enhancement was detected with other combination, which aligns with the morphological changes in AuS observed exclusively in the presence of HH and Au precursor (Figure S5c, Supporting Information). This indicates that the Raman signal enhancement is a result of the Au reduction process, which leads to molecular entrapment. To further validate the role of AuS, we conducted additional experiments to confirm its effect on Raman enhancement during PME (Figure S6a,b, Supporting Information). Strong Raman enhancement was observed only in the presence of AuS, while no signal enhancement was detected in its absence, confirming its role as both a nucleation site and a molecular adsorption site.

To determine the optimal PME conditions for molecular detection, various Au precursor to HH ratios (ranging from 1:0.5 to 1:10) were analyzed using Raman spectroscopy (Figure S7a,b, Supporting Information) and SEM imaging (Figure S8, Supporting Information). At a low ratio of 1:0.5, weak Raman signal enhancement was observed, which correlated with SEM images showing sparse protrusions on the AuS surface. This limited structural evolution resulted from an insufficient amount of HH, which was unable to effectively reduce Au ions.^[^ ^39^ ^]^ As the HH increased, a greater number of protrusions formed, leading to a progressive increase in Raman intensity, with the highest enhancement observed at a ratio of 1:2. However, at ratios exceeding 1:2, SEM analysis revealed excessive particle growth and aggregation, which obstructed hotspot sites, thereby reducing the overall enhancement. Based on these findings, the 1:2 ratio was determined to be the optimal condition for PME in molecular detection. To further refine the conditions, various concentrations of Au precursor and HH at the same optimized ratio (ranging from 10 mm: 20 mm to 50 mm:100 mm) were investigated (Figure S9a,b, Supporting Information). The Raman signal was gradually increased, peaking at 40 mm: 80 mm, and then decreased with increasing concentration. This signal evolution is likely due to geometric changes in the nucleation site. The SEM image revealed the enlargement of protrusions, with measured widths of 138.9 ± 31.4 nm, 191.6 ± 31.9 nm, 224.2 ± 38.6 nm, 244.6 ± 42.1 nm, and 262.2 ± 50.7 nm for concentration of 10 mm: 20 mm, 20 mm: 40 mm, 30  mm: 60 mm, 40 mm: 80 mm, and 50 mm:100 mm, respectively (Figure S10a,b, Supporting Information). This suggests that increasing protrusion size enhances the Raman signal due to the generation of hotspot. However, concentrations greater than 40 mm: 80 mm led to a reduction in the Raman signal, attributed to obstructed light penetration into the hotspot regions due to the formation of a thick AuNL layer.

To investigate the SERS performance of the optimized PME method, various concentrations of 4‐ATP solution at 10^−7^–10^−13^ m were tested under laser excitation of 785 nm for 10 s after 2 min of PME treatment. The 4‐ATP solution was detected down to 10^−13^ m with the calculated LOD of 1.9 × 10^−14^ m (Figure 2g). The standard curves obtained for the characteristic peak of 4‐ATP at 1 071 cm^−1^ showed linear correlations within the concentration range of 4‐ATP with high R^2^ value (Figure S11a, Supporting Information). The enhancement factor (EF) was calculated after measurement of 4‐ATP solution on the bare silicon wafer and PME. The EF was determined to be 5.3 × 10^8^, indicating a high potential for use in Raman measurement methods (Figure S11b, Supporting Information). In addition, the developed PME method was evaluated for uniformity of molecular sensing. Raman signals of 4‐ATP showed a uniform distribution over 4 µm × 4 µm mapping area (Figure S11c, Supporting Information). The SERS intensities at the characteristic peak of 1 071 cm^−1^ were obtained from 100 different points, displaying uniform Raman signal generation with relative standard deviation (RSD) values of 8.95%. Batch‐to‐batch signal uniformity of PME also tested 10 times, demonstrating reproducible method with RSD values of 7.26% (Figure S11d, Supporting Information). Therefore, our developed PME method exhibits the potential to be effectively used for various sensing field applications.

### 2.2 PME for Label‐Free DNA Detection

**Figure 3:**
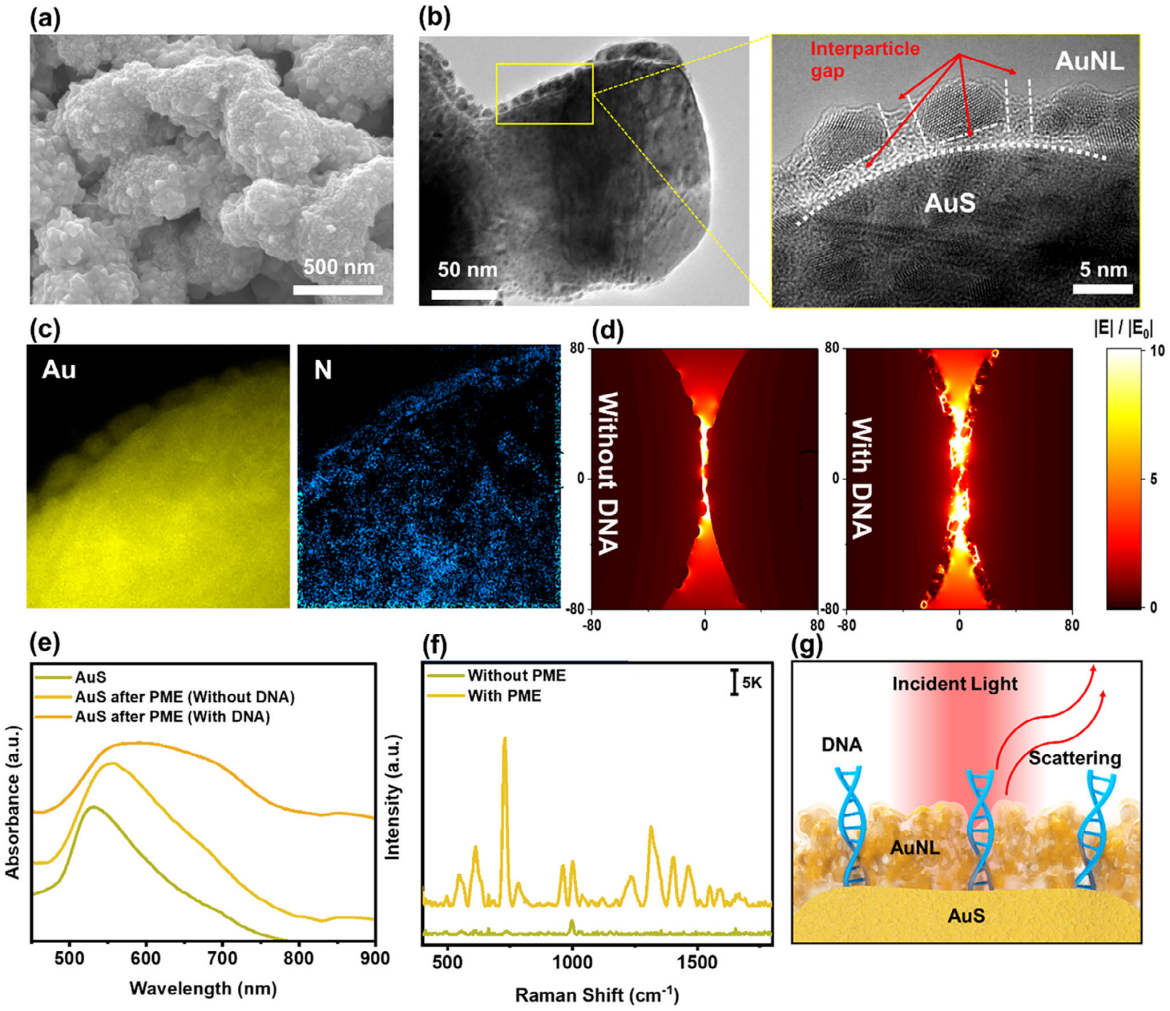
Material characterization of PME method in the presence of target DNA. a) SEM image of AuS after PME, b) TEM (left) and high resolution‐TEM (right) images of the laminated area on AuS surface, c) elemental mapping on the laminated area, d) electromagnetic field distribution of PME, e) UV–vis absorbance spectra and f) SERS enhancement of PME method for DNA detection (scale bar refers to SERS intensity), g) Raman signal enhancement mechanism of PME method.

To evaluate the capability of the PME method to differentiate DNA nucleobases, 100 µL of customized DNA at a concentration of 100 ng mL⁻^1^, dissolved in diethyl pyrocarbonate (DEPC) water, was analyzed using the optimized trapping agent ratio (HAuCl_4_:HH = 1:10) and reaction time (t = 10 min) (Figure S12a,b, Supporting Information). The morphological characteristics of the PME method in the presence of DNA were investigated through SEM analysis of AuS. Notably, the AuS surface underwent significant morphological evolution in the presence of DNA. As shown in **Figures** **3**a and S13 (Supporting Information), AuNL with multispike protrusions densely formed on the AuS surface. This transformation is attributed to the competitive interaction of nucleotides in the DNA strand with Au atom during the shaping process, leading to a roughened AuS surface, as reported elsewhere.^[^ ^47^, ^48^, ^49^ ^]^ To further explore the in situ growth mechanism of AuNL, the HR‐TEM was conducted on the boundary regions of the AuNL layer. As shown in Figure 3b and Figure S14 (Supporting Information), interparticle gaps of 2.52 ± 0.73 nm were observed on the AuNL layer, suggesting that DNA modulates the hotspot site generation. Adsorbed DNA on the nucleation site of AuS appeared to hinder the growth rate on the specific crystal facet, stabilizing these regions and promoting the formation of interparticle gaps and multispike protrusions.^[^ ^49^, ^50^, ^51^, ^52^ ^]^ To further confirm the trace of DNA on the AuNL layer, electron energy loss spectroscopy (EELS) elemental mapping was performed on the TEM‐imaged area (Figure 3c). Nitrogen mapping revealed multiple nitrogen sites on the AuS surface, consistent with nucleotides as nitrogen‐containing biological compounds,^[^ ^53^ ^]^ confirming the uniform distribution of DNA on the AuS surface. This observation supports the proposed DNA entrapment mechanism of the PME method.

The electric field (E‐field) distribution surrounding plasmonic nanomaterials is crucial for generating strong SERS signals. To verify the activation of hotspot regions on the AuS surface induced by the PME method, computational simulations were conducted using a finite‐difference time‐domain (FDTD) model, both in the absence and presence of DNA. The FDTD model was designed to replicate the observed structure from SEM and TEM images. Simulation parameters included polarized incident light (785 nm) directed vertically onto the AuS surface, focusing on interparticle gaps within the AuNL regions. As shown in Figure 3d, intense hotspots are generated at interstructural positions of the AuS, regardless of the presence of DNA. Interestingly, in the presence of DNA, interior hotspot emerges within the AuNL region due to interparticle gaps around the adsorbed DNA, resulting in a concentrated E‐field distribution within this area. The z‐axis position‐dependent E‐field distribution images, both without and with DNA, are presented in Figures S15 and S16 (Supporting Information), respectively. At the same z positions, the intensity of the E‐field at the AuNL region is significantly enhanced with DNA compared to without DNA. The in situ real‐time E‐field simulation videos of PME (Videos S1 and S2, Supporting Information) confirm interior hotspot formation inside the AuNL region in the presence of DNA. These results suggest that the PME method effectively generates localized hotspot regions around the target analyte, providing substantial Raman scattering enhancement.

To further examine the optical properties of the PME method, UV–vis absorbance measurement was performed on the AuS substrate before and after PME, both in the absence and presence of DNA (Figure 3e). Without DNA, the spectrum showed a shift in the SPR peak from 525 to 560 nm, indicating an increase in particle size due to the deposition of an additional AuNL layer on the AuS surface.^[^ ^37^ ^]^ In contrast, when DNA was present, a broad SPR peak spanning from 500 to 800 nm was observed. This broadening reflects significant morphological evolution, attributed to the formation of multispike protrusions.^[^ ^52^ ^]^ The extended SPR range suggests improved light absorption and scattering properties, making longer laser wavelength potentially more effective for Raman measurement.^[^ ^37^ ^]^ To evaluate the SERS performance of PME, we performed Raman analysis of DNA in both of without and with PME (Figure 3f). Without PME, only DNA components with strong adsorption and high polarizability,^[^ ^45^, ^46^ ^]^ such as guanine (612 cm^−1^), adenine (726 cm^−1^), deoxyribose sugar (1000 cm^−1^), and phosphate backbone (1048 cm^−1^),^[^ ^47^, ^48^, ^49^ ^]^ were detected. In contrast, PME enhances all vibrational modes (Table S3, Supporting Information), including cytosine (782 cm^−1^) and thymine (1233 cm^−1^), which have relatively lower Raman cross‐section, making previously weak or undetectable Raman peaks visible. This enhancement arises from multiple factors, including the formation of localized hotspot around DNA targets, plasmonic coupling between adjacent nanostructures, and edge effects. These mechanisms collectively amplify Raman scattering, leading to a significant increase in Raman signal intensity (Figure 3g).

### 2.3 SERS Performance for DNA Profiling

**Figure 4:**
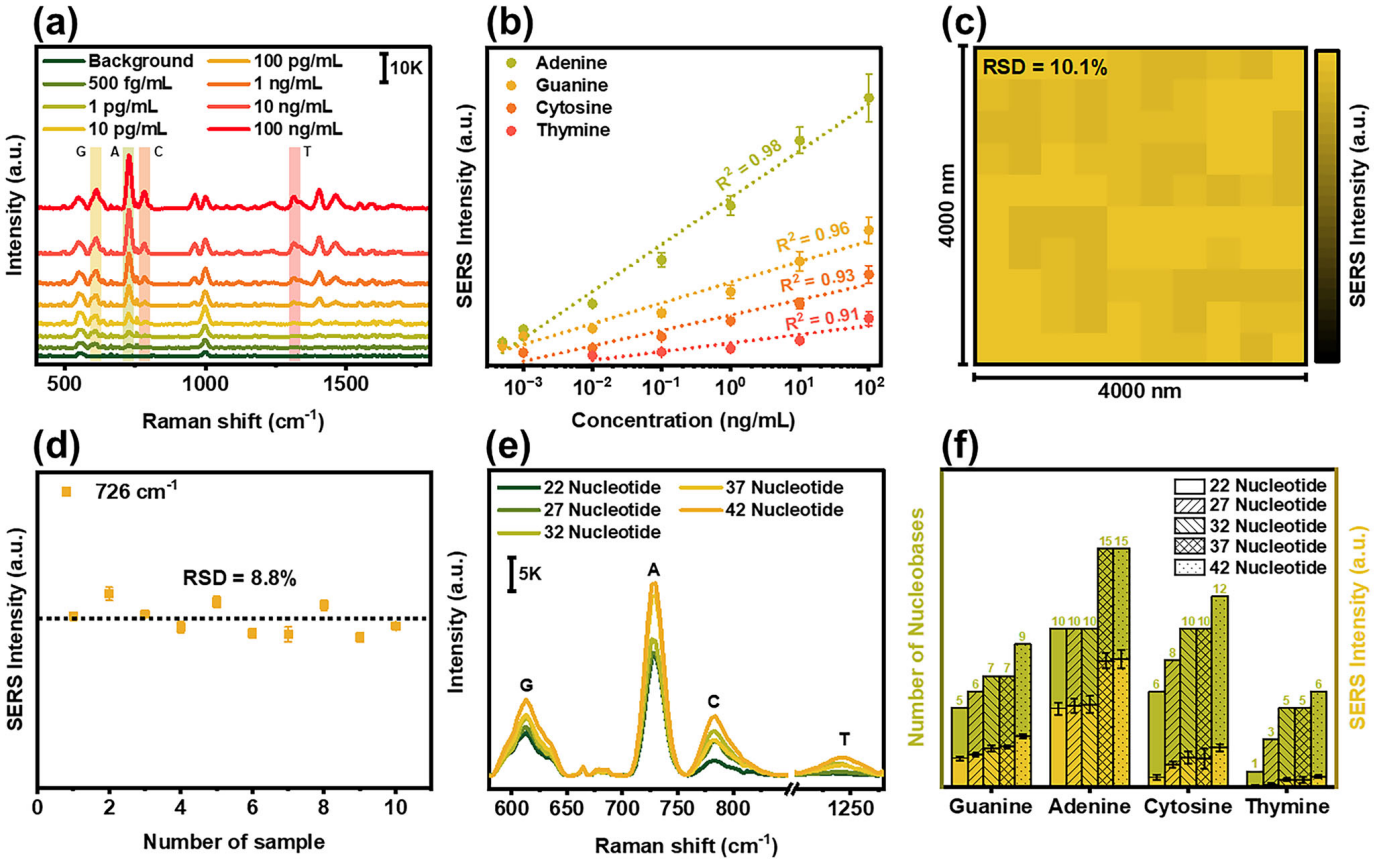
SERS performance of PME method for DNA profiling. a) SERS performance of the PME method at various DNA concentrations, b) standard curves for quantitative analysis of DNA, c) signal uniformity test with 100 different points, d) reproducibility test with ten independent tests, e) SERS spectra (scale bar refers to SERS intensity) and f) signal intensity analysis of various DNA length.

The SERS performance of the optimized PME method was investigated using 100 µL of customized DNA under a laser excitation wavelength of 785 nm. To investigate the sensitivity of PME method for DNA detection, various DNA concentrations ranging from 100 ng mL⁻^1^ to 500 fg mL⁻^1^ were tested. As shown in **Figure** **4**a, DNA components of guanine (612 cm^−1^), adenine (726 cm^−1^), cytosine (782 cm^−1^), and thymine (1 233 cm^−1^) were detected at different limits of detection, with calculated LOD values of 25.6 fg mL⁻^1^, 23.6 fg mL⁻^1^, 346 fg mL⁻^1^, and 618 fg mL⁻^1^, respectively. This trend suggests that Raman cross‐section strongly influences detection sensitivity, as adenine exhibits the highest Raman cross‐section, followed by guanine, cytosine, and thymine, leading to progressively lower sensitivity for these nucleobases. The standard curve for the characteristic peaks of adenine, guanine, cytosine, and thymine exhibited linear correlations across the tested DNA concentrations, with R^2^ value of 0.98, 0.96, 0.93, and 0.91, respectively (Figure 4b). Furthermore, the uniformity and reproducibility of the SERS signal are critical factor for determining the reliability of a SERS technique. Raman mapping was performed over a 4 µm × 4 µm area with 100 different measurement points, revealing a consistent Raman signal with an RSD of 10.1% (Figure 4c). Additionally, batch‐to‐batch signal uniformity of the PME method was confirmed through ten independent tests, yielding an RSD value of 8.8% (Figure 4d). Furthermore, to evaluate the DNA profiling capabilities of PME, DNA sequences of varying lengths (22 to 42 nt; Table S4, Supporting Information) were analyzed. Figure 4e presents the representative SERS spectra of these DNA samples, highlighting distinct peak patterns in regions corresponding to guanine (550–650 cm^−1^), adenine (700–750 cm^−1^), cytosine (750–820 cm^−1^), and thymine (1200–1270 cm^−1^). Notably, the SERS intensity of these peaks increased proportionally with the number of corresponding nucleobases in each sequence and was further modulated by their intrinsic Raman cross‐sections (Figure 4f). In sequence with an equiv. number of nucleobases, adenine exhibited a significantly higher Raman intensity than cytosine, and similar trends were observed for guanine and thymine. These findings indicated that the Raman cross‐section strongly influenced the SERS signal dynamics in DNA strands, demonstrating the capability of PME method for detailed DNA profiling.

### 2.4 PME for DNA Methylation Quantification

**Figure 5:**
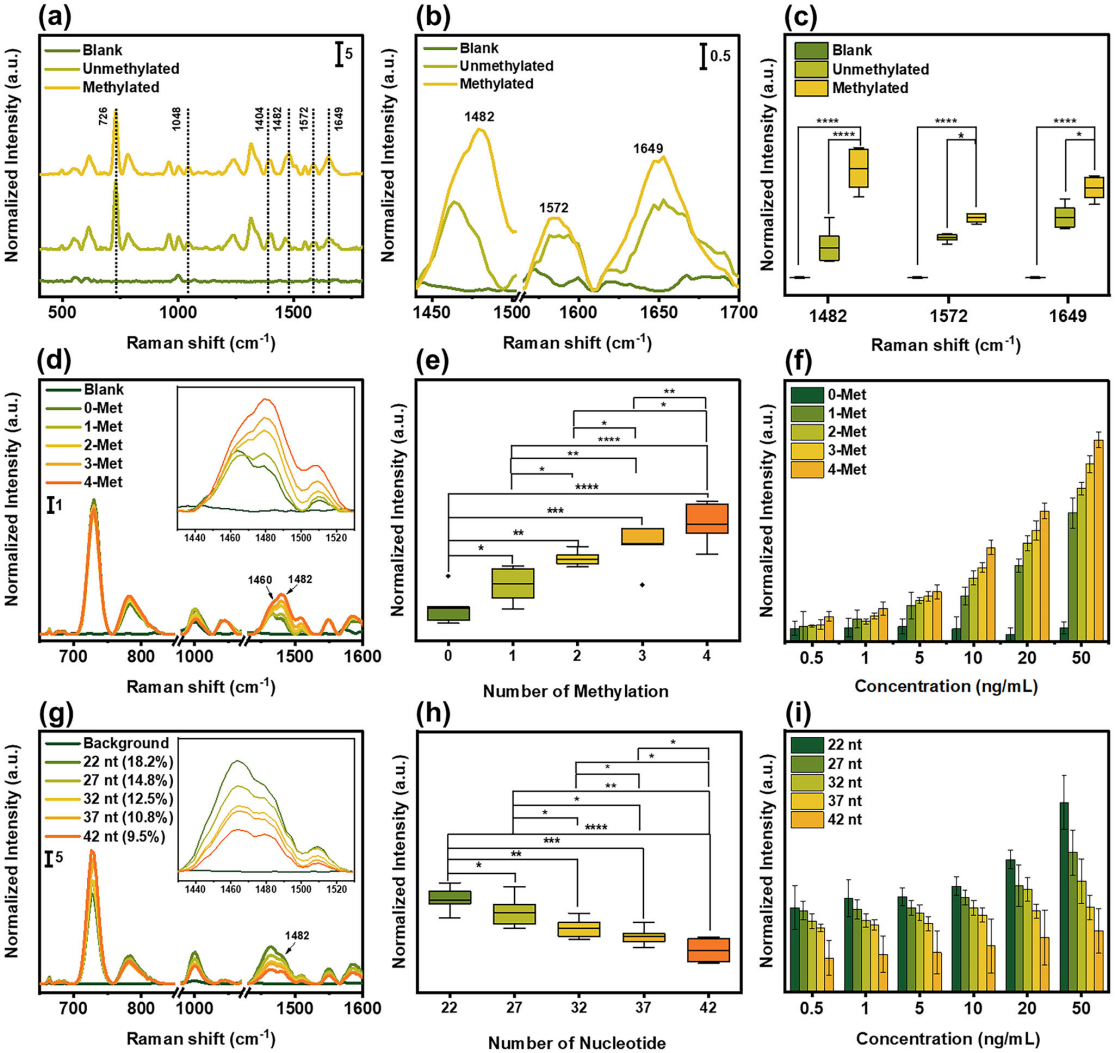
The PME method for DNA methylation quantification analysis. a) SERS spectra of unmethylated and methylated DNA (scale bar refers to normalized SERS intensity), b) SERS spectral markers for DNA methylation, and c) SERS intensity comparison of blank (*n* = 10), unmethylated (*n* = 10), and methylated DNA (*n* = 10) at methylation marker peaks using one‐way ANOVA followed by Tukey's post‐hoc test (*p < 0.05, **p < 0.01, ***p < 0.001, and ****p < 0.0001). Methylation degree analysis d) SERS spectra and magnification band at 1 430 to 1 530 cm^−1^ (inset) (scale bar refers to normalized SERS intensity), e) Raman intensity comparison performed using one‐way ANOVA followed by Tukey's post‐hoc test (*n* = 10 per groups), and f) concentration dependent analysis at methylation marker of 1 482 cm^−1^. DNA size analysis (error bar refers to standard deviation), g) SERS spectra and magnification band at 1 430–1 530 cm^−1^ (inset) (scale bar refers to normalized SERS intensity), h) Raman intensity comparison performed using one‐way ANOVA followed by Tukey's post‐hoc test (*n* = 10 per groups), and i) concentration dependent analysis at methylation marker (error bar refers to standard deviation).

After establishing PME for label‐free DNA sensing, we applied our method to quantify DNA methylation. We first examined 100 µL of both unmethylated and methylated DNA at concentration of 100 ng mL⁻^1^. The final methylation concentration was calculated to be 18.2 ng mL⁻^1^, corresponding to 18.2% methylated nucleotides within the samples. As shown in **Figure** **5**a, Raman spectra of unmethylated and methylated DNA exhibit distinct peaks in the range of 400–1 800 cm^−1^, corresponding to deoxyribose (1404 cm^−1^), a phosphate group (1048 cm^−1^), a nucleobase (726 cm^−1^), and a methyl group (1482 cm^−1^). To accurately quantify the spectral variations induced by methylation, all spectra were normalized to the ν(PO_2_^−^) peak at 1 048 cm^−1^, as this feature remains consistent regardless of changes in the DNA condition (Figure S17, Supporting Information).^[^ ^17^ ^]^ Among the numerous DNA characteristic peaks, three distinct peaks were identified at 1482, 1572, and 1649 cm^−1^, exhibiting prominent positive bands specifically in methylated DNA (Figure 5b).^[^ ^23^, ^24^, ^54^ ^]^ These peaks were considered as primary spectral markers for DNA methylation due to their clear intensity changes and shifts upon methylation, reflecting structural modifications.^[^ ^17^ ^]^ To further confirm the differences in the Raman spectra among blank, unmethylated, and methylated DNA, we compared the Raman intensities at these methylation marker peaks and conducted statistical analysis using one‐way ANOVA followed by Tukey's post‐hoc test. The results showed a significant increase in methylation peak intensities in the methylated DNA samples compared to controls, with a *p*‐value of less than 0.05 (Figure 5c). These findings demonstrate that PME method can sensitively and precisely detect methyl groups at cytosine‐phosphate‐guanosine (CpG) sites by identifying specific methylation marker peaks.

Recent studies have shown that global hypo‐ and hypermethylation is epigenetic markers for identifying cancer stem cells.^[^ ^1^, ^2^ ^]^ As a result, quantitative analysis of DNA methylation levels is critical for the diagnosis and post‐treatment monitoring of methylation‐related diseases.^[^ ^9^ ^]^ To further elucidate the correlation between methylation level and Raman signal intensity, we first analyzed DNA samples with varying number of methyl groups (0–4) at CpG sites, as listed in Table S5 (Supporting Information). The Raman spectral features of these groups showed significant differences, particularly in cytosine (1 460 cm^−1^) and methylation marker (1482 cm^−1^) peaks (Figure 5d). Interestingly, in smaller methylation degree, only cytosine peak was detected whereas increasing the methylation number at CpG sites allowed for the detection of both the methylation marker and the cytosine peak. To further explore this observation, we compared the Raman intensity of the methylation marker peaks across different methylation degree (Figure 5e). The data showed an increase in the methylation marker peak with increasing methylation degree. This result correlates with the number of methylation groups in the CpG sites, leading to structural modifications.^[^ ^3^, ^55^ ^]^ To investigate the influence of total DNA methylation concentration on Raman intensity across different methylation levels, we tested a concentration series of methylation level ranging from 50 to 0.5 ng mL⁻^1^. The results showed an exponential relationship between the methylation marker peak intensity and the concentration for all methylation levels (Figure 5f; Figure S18, Supporting Information). This behavior can be attributed to the influence of methylation on DNA binding behavior and conformation,^[^ ^55^, ^56^ ^]^ which leads to localized clustering of methylated regions and results in cooperative electromagnetic enhancement, where the Raman signal increases non‐linearly.^[^ ^57^ ^]^ We further explored the correlation between methylation ratio and Raman intensity by analyzing DNA lengths ranging from 22 to 42 nt, each containing the same number of methyl groups, as specified in Table S6 (Supporting Information). The Raman spectral patterns of the five different groups showed distinct differences, particularly in the methylation marker peak at 1 482 cm^−1^ (Figure 5g). The Raman intensity comparison at this peak decreased proportionally with the DNA length (Figure 5h). This result correlates with number of nucleotide and methyl group in the promoter area, reflecting the reduction of methyl groups ratio with lengthening the DNA. Additionally, we evaluated the correlation between total DNA methylation concentration on Raman intensity across different methylation ratios, with concentrations ranging from 50 to 0.5 ng mL⁻^1^. The results also demonstrated an exponential relationship between the methylation marker peak and concentration for all methylation ratios, with a general decrease in overall intensity as the methylation ratios decreased (Figure 5i; Figure S19, Supporting Information). These findings demonstrate the high sensitivity and specificity of the PME method for detecting DNA methylation, presenting a promising technique for DNA methylation quantification.

### 2.5 Machine Learning‐Assisted DNA Methylation Quantification in Human Serum

**Figure 6:**
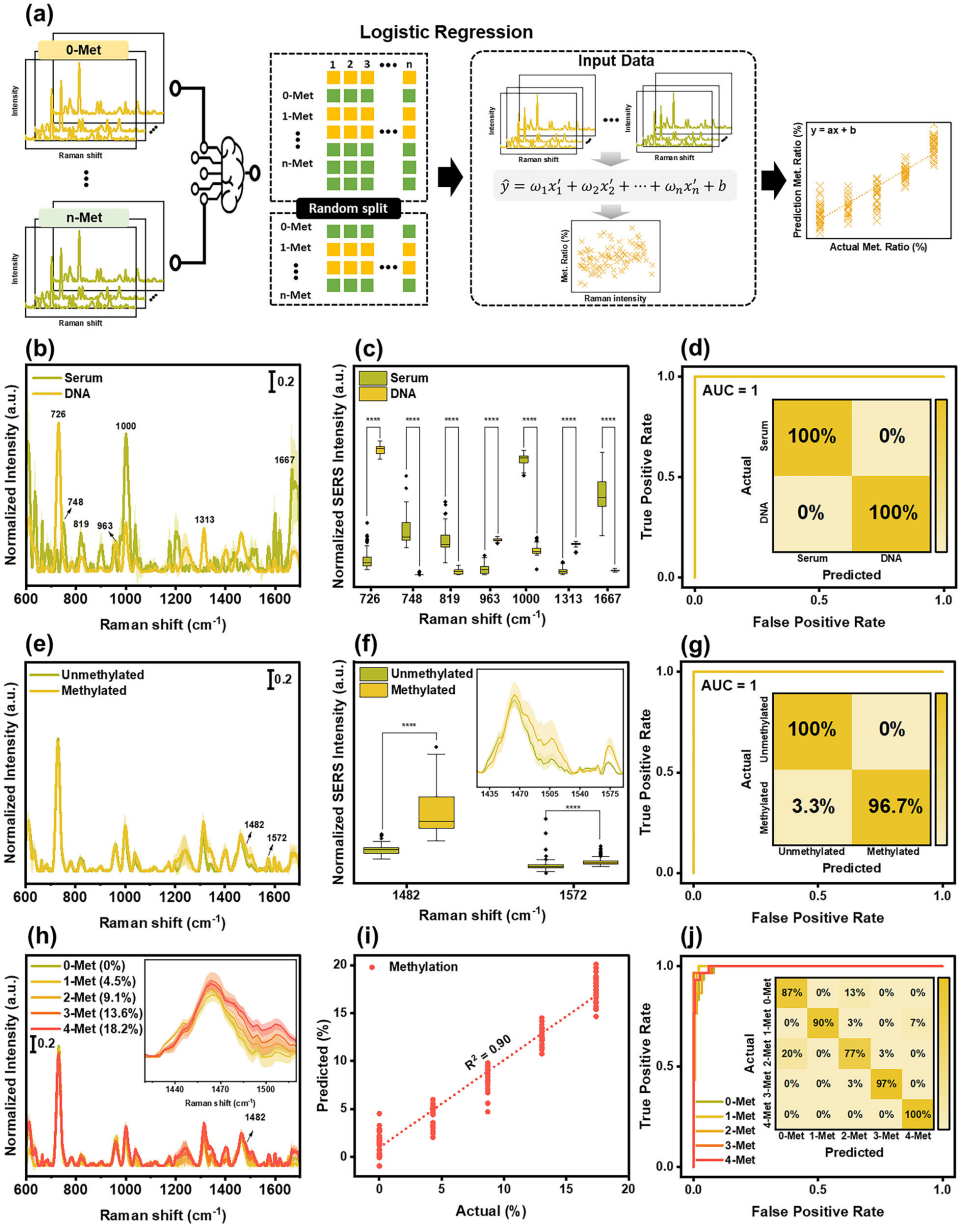
DNA methylation quantification and classification in human serum. a) schematic illustration of LR‐assisted machine learning model, b) SERS spectra of human serum and DNA in serum (scale bar refers to normalized SERS intensity), c) selected Raman peak and intensity comparison of human serum (*n* = 100) and DNA (*n* = 100) using *t*‐test (*p < 0.05, **p < 0.01, ***p < 0.001, and ****p < 0.0001), d) ROC curve and confusion matrix (inset) of human serum and DNA, e) SERS spectra of unmethylated and methylated DNA in human serum (scale bar refers to normalized SERS intensity), f) Raman intensity comparison using *t*‐test and magnification of selected Raman peak (inset) of unmethylated (*n* = 100) and methylated (*n* = 100) DNA, g) ROC curve and confusion matrix (inset) of unmethylated and methylated DNA, h) SERS spectra of various methylation levels and magnification of selected Raman peak (inset) (scale bar refers to normalized SERS intensity), i) quantification of global DNA methylation levels in human serum, j) ROC curve and confusion matrix (inset) of various methylation levels.

The feasibility of the PME method for DNA methylation analysis was demonstrated using human serum samples, which holds clinical significance for early detection and monitoring of disease such as cancer.^[^ ^58^ ^]^ In this study, both unmethylated and methylated DNA derived from a colon cancer‐specific sequence^[^ ^28^ ^]^ were spiked into 100 µL human serum to simulate clinical conditions, achieving a final DNA concentration of 10 ng mL⁻^1^ and a methylation concentration of 1.82 ng mL⁻^1^, as previously reported.^[^ ^23^ ^]^ To precisely classify and quantify the Raman spectra of different DNA groups in human serum, machine learning‐based LR analysis was applied. LR was selected for its simplicity and interpretability, as it provides direct insight into the contribution of individual features through model coefficients.^[^ ^59^, ^60^ ^]^ This enables the correlation of specific Raman shifts with DNA methylation levels in a biologically meaningful manner. The model workflow included data processing, model training, prediction, and evaluation (**Figure** **6**a), allowing for the establishment of a quantitative relationship between Raman intensity and methylation ratio. The LR was trained by fitting a linear equation of the 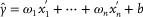, where the $\hat{y}$ is the predicted methylation ratio, $x_{1}^{\prime },x_{2}^{\prime },\text{…},x_{n}^{\prime }$ are the standardized Raman intensities at selected shifts, and $\omega_{1},\omega_{2},\text{…},\omega_{n}$ are the LR coefficients that determine the contribution of each Raman shift to the prediction, and *b* is the intercept.

First, PME was performed on human serum and spiked DNA to investigate their Raman spectral behavior (Figure 6b). Distinct Raman spectral patterns were observed for human serum and DNA, showing clear spectral differences particularly in the regions of 600–820 cm^−1^, 850–1100 cm^−1^, and 1150–1700 cm^−1^. To determine the critical Raman peak positions contributing to spectral discrimination, the LR model was applied. A two‐step statistical analysis was performed to identify significant spectral features: a *t*‐test with a cutoff *p*‐value < 0.05 was first applied to detect statistically significant Raman peaks, followed by a partial least square regression (PLSR) analysis with a variable importance in projection (VIP) score cutoff  > 1.4 to further refine the selection of Raman shifts relevant for classification. Figure 6c shows the selected Raman peak positions with the highest statistical significance. As expected, the human serum spectra were dominated by vibrational modes of albumin (748 cm^−1^, 819 cm^−1^, and 1000 cm^−1^) and globulin (1667 cm^−1^), the two predominant serum proteins.^[^ ^23^, ^61^ ^]^ Upon addition of DNA, the Raman spectra exhibited dominant adenine peaks (726 cm^−1^), along with features corresponding to other nucleobases (1313 cm^−1^) and deoxyribose sugar (963 cm^−1^) with minimal interference from serum components. This phenomenon arises from the strong binding affinity of DNA to the Au surface, attributed to multiple nitrogenous bases, π‐electron interactions, and phosphate‐Au coordination.^[^ ^47^, ^62^ ^]^ In contrast, albumin and globulin primarily interact with Au surface through limited thiol (‐SH) groups and weak Au‐imidazole binding from histidine residues, leading to the dominant DNA signal in serum samples due to competitive adsorption.^[^ ^63^, ^64^ ^]^ The large and complex tertiary structure of proteins also limits their surface accessibility to Au, reducing their affinity compared to DNA.^[^ ^65^ ^]^ In addition, the Raman cross‐section of serum proteins is relatively low compared to DNA strand,^[^ ^66^ ^]^ enabling clear discrimination of DNA peaks. This competitive adsorption of DNA onto the Au surface in human serum highlights the capability of PME for efficient DNA detection, even in complex biological matrices. This ability is critical for clinical applications, where accurate detection of DNA is necessary for signaling early‐stage disease. To further validate the diagnostic accuracy of PME, the LR model was applied for the classification of DNA from human serum. The dataset was randomly divided into 70% training and 30% test dataset, after collecting 100 Raman spectra of each human serum and DNA samples. The model achieved perfect accuracy, with 100% sensitivity and specificity for all groups, as demonstrated in confusion matrix (Figure 6d, inset). The receiver operating characteristics (ROC) curves also yielded an area under the curve (AUC) of 1 (Figure 6d), confirming excellent classification performance.

PME was further applied to unmethylated and methylated DNA in human serum to investigate their Raman spectral behavior. The Raman spectral patterns of unmethylated and methylated DNA appeared similar (Figure 6e; Figure S20, Supporting Information), making visual differentiation more challenging. Therefore, the LR model was applied to identify critical Raman peak position using the previously described two‐step statistical analysis. Figure 6f shows the selected Raman peak positions with magnified views of the methylation marker bands at 1 482 and 1 572 cm^−1^, which is exhibited the highest statistical significance. These marker bands correspond to vibrational modes associated with C‐H bending and aromatic ring stretching, which are known to be influenced by epigenetic methylation modifications. Their prominence in the statistical analysis suggests that methylation‐induced structural changes in DNA contribute to distinct Raman signatures, enabling reliable discrimination between methylated and unmethylated states. To further validate the diagnostic accuracy of PME for DNA methylation detection, the LR model was applied classify methylated DNA from unmethylated DNA. The confusion matrix results revealed sensitivity of 96.7%, specificity of 100%, and accuracy of 98.3% (Figure 6g, inset). The ROC curves also showed AUC value of 1 (Figure 6g), demonstrating the high classification performance of PME for DNA methylation detection. In addition, the LOD for methylated DNA in human serum was found to be 0.5 ng mL⁻^1^ (Figure S21, Supporting Information), underscoring the high sensitivity of this approach. As DNA in clinical samples such as plasma commonly degraded to small fragments and is present at only few nanograms per milliliter.^[^ ^7^ ^]^ This low LOD is significant in clinical diagnostics because it enables the detection of small amounts of circulating methylated DNA, which is crucial for early disease detection, particularly in cancers where DNA methylation serves as biomarker.^[^ ^7^, ^10^ ^]^

We further extended this approach to analyze DNA samples with varying global methylation levels (0%, 4.5%, 9.1%, 13.6%, and 18.2%) in human serum, reflecting the complexity of real clinical scenarios where DNA may exhibit different degree of methylation. Detecting variations in global methylation level is crucial for diagnosing and monitoring diseases such as cancer, as abnormal methylation patterns are often associated with gene silencing and tumor progression.^[^ ^4^ ^]^ The representative SERS spectra for different methylation degree are shown in Figure 6h. Among them, methylation marker peak at 1 482 cm^−1^ was selected for further investigation in quantification analysis. The LR model was implemented to quantify global DNA methylation levels by fitting a linear equation that converts Raman intensity into a predicted methylation ratio, representing the estimated percentage of methylated DNA in a sample. The model achieved a mean absolute error (MAE) of 3.58 and an R^2^ of 0.90 (Figure 6i), demonstrating high predictive accuracy. The MAE reflects the average deviation between predicted and actual methylation levels, while the strong R^2^ value confirms a clear correlation between Raman intensity and global methylation levels, highlighting the reliability of this approach. These results highlight the capability of PME to quantitatively assess global DNA methylation with high precision, offering a promising approach for label‐free detection in clinical application. We further applied the LR model to classify global DNA methylation levels. The method successfully distinguished different methylation states with an overall classification accuracy of 90%, highlighting its potential diagnostic capability. The confusion matrix results reveal sensitivities of 86.7%, 90%, 76.7%, 96.7%, and 100% and specificities of 95%, 100%, 95%, 99.2%, and 98.3% for methylation ratios of 0%, 4.5%, 9.1%, 13.6%, and 18.2%, respectively (Figure 6j, inset). The ROC curves for these categories yielded AUC values of 0.99, 1, 0.99, 1, and 1, respectively (Figure 6j), further confirming the robust performance of this method for detailed DNA methylation analysis, with significant implications.

### 2.6 Clinical Applications

The clinical applicability of PME for DNA methylation quantification was evaluated using 60 serum samples from patients with colorectal cancers (CRC) (*n* = 40) and normal individuals (*n* = 20) from Samsung Medical Center, South Korea. The clinical characteristics of the patients are summarized in Tables S7 (Supporting Information). The histological type and cancer stage were confirmed using the clinically established carcinoembryonic antigen (CEA) test, with CRC cases further classified into stage I (*n* = 10), stage II (*n* = 10), stage III (*n* = 10), and stage IV (*n* = 10). PME was directly applied to 100 µL of patient serum samples without any pre‐treatment, followed by methylation analysis using the previously trained model (Figure 6a). Notably, the ability of PME to analyze raw serum samples without complex processing underscores its potential as a rapid and accessible diagnostic tool in clinical field.

**Figure 7:**
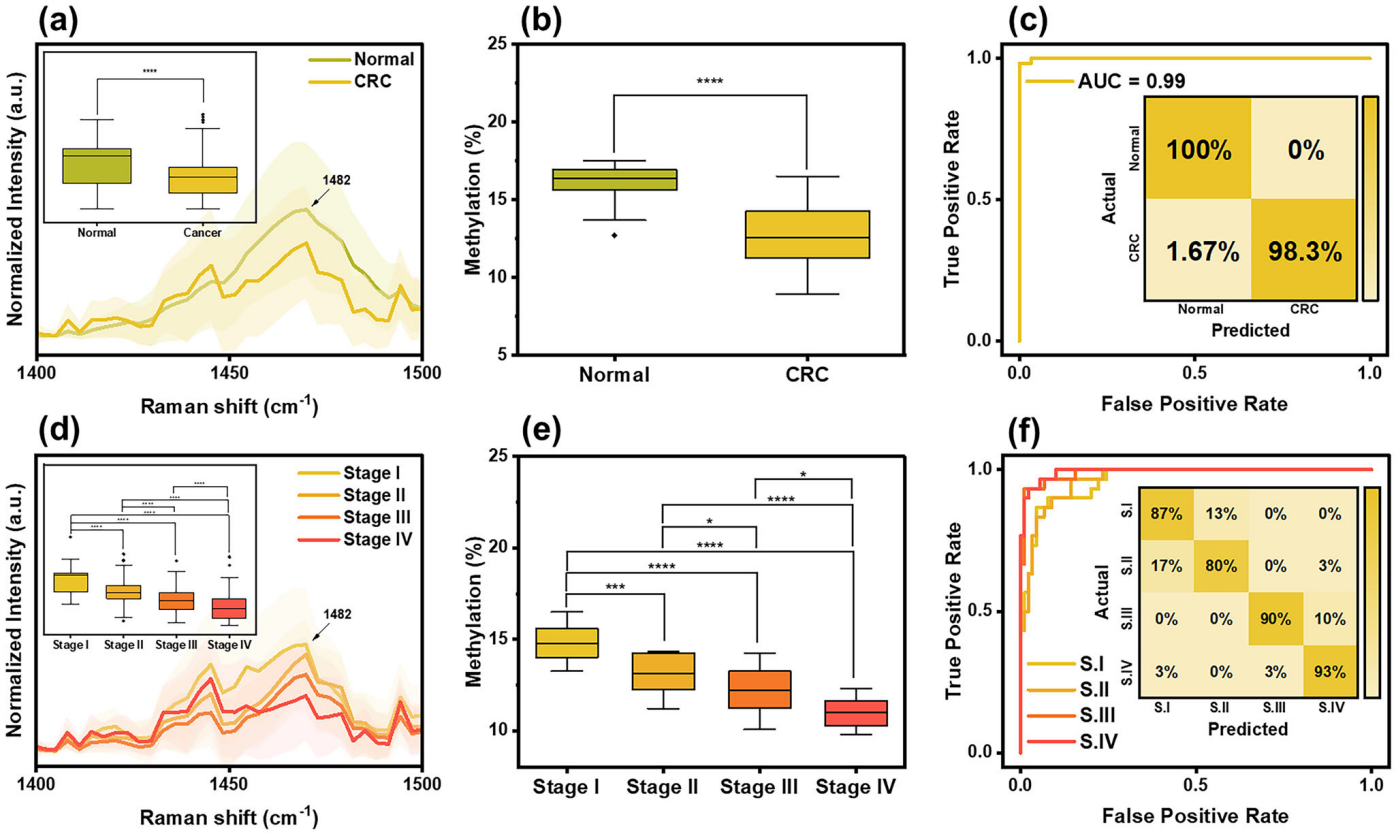
Clinical application. a) SERS spectra and intensity comparison at 1482 cm^−1^ (inset) of clinical serum from normal (*n* = 200) and CRC (*n* = 400) samples using *t*‐test (*p < 0.05, **p < 0.01, ***p < 0.001, and ****p < 0.0001), b) methylation prediction comparison of normal (*n* = 200) and CRC (*n* = 400) samples using *t*‐test, c) ROC curve and confusion matrix (inset) of normal and CRC patients, d) SERS spectra and intensity comparison at 1482 cm^−1^ (inset) of CRC stages using one‐way ANOVA followed by Tukey's post‐hoc test (*n* = 100 per groups), e) methylation prediction comparison of CRC stages using one‐way ANOVA followed by Tukey's post‐hoc test (*n* = 100 per groups), and f) ROC curve and confusion matrix (inset) of CRC stages patients.

First, PME was performed on normal and CRC samples to investigate their Raman spectral behavior. Raman spectra were collected after 10 min of PME reaction. **Figure** **7**a and Figure S22a (Supporting Information) present the averaged SERS signals obtained from normal and CRC samples. The representative SERS spectra from both groups exhibited similar spectral profiles, with a significant difference observed at 1 482 cm^−1^, corresponding to C‐H bending (Figure 7a, inset; Figure S22b, Supporting Information). This peak was selected to quantify global methylation levels using an LR‐based model, leveraging Raman intensity variations. The results revealed lower methylation levels in CRC samples (12.7 ± 2.1%) compared to normal samples (15.9 ± 1.3%) (Figure 7b; Figure S23, Supporting Information), which is associated with global hypomethylation.^[^ ^5^, ^67^, ^68^ ^]^ This phenomenon has been clinically reported and linked to the loss of methylation in repetitive elements, DNA methyltransferase (DNMT) dysfunction, DNA demethylation activity, and chromosomal instability.^[^ ^68^, ^69^, ^70^ ^]^ Further classification analysis using the previously trained LR model between these groups demonstrated sensitivity, specificity, and accuracy of 100%, 98.3%, and 99.2%, respectively (Figure 7c, inset). To evaluate the robustness of this model for limited clinical datasets, fivefold cross‐validation was conducted using corresponding Raman spectral features from all groups. The model maintained consistently high performance, with fold‐wise accuracies ranging from 98.8% to 100% confirming its reliability in classifying CRC from normal samples (Figure S24a, Supporting Information). The ROC curve yielded and AUC of 0.99 (Figure 7c), highlighting the strong diagnostic potential of PME for clinical applications.

Given this potential, we further investigate PME for cancer stage analysis, as early cancer detection is crucial for timely intervention and improved patient outcomes. Figure 7d and Figure S22a (Supporting Information) present the averaged SERS signals obtained from each CRC stage group, including stages I, II, III, and IV. Similar Raman spectral profiles were observed across all stages, with a gradual decrease in the methyl marker peak intensity at 1 482 cm^−1^ as cancer progressed (Figure 7d, inset; and Figure S22b, Supporting Information). This trend is attributed to increasing genomic instability, cumulative DNA damage, and epigenetic dysregulation as clinically reported.^[^ ^67^, ^71^ ^]^ Consistently, the global methylation level predictions (Figure 7e; Figure S23, Supporting Information) revealed a progressive decline with advancing cancer stage: stage I (14.4 ± 2.1%), stage II (13.1 ± 1.1%), stage III (12.3 ± 2.4%), and stage IV (11.2 ± 0.9%). Multi‐class classification successfully distinguished different CRC stages with an overall accuracy 87.5%, highlighting diagnostic capability of PME. The confusion matrix results further demonstrate sensitivities of 86.7%, 80%, 90%, and 93.3% and specificities of 93.3%, 95.5%, 98.9%, and 95.6% for stage I, II, III, and IV, respectively (Figure 7f, inset). The fivefold cross‐validation also showed consistently high performance, with fold‐wise accuracies ranging from 81.5% to 93.8%, confirming the robustness of LR model in distinguishing CRC stages (Figure S24b, Supporting Information). Additionally, the ROC curves yielded AUC values of 0.96, 0.96, 0.98, and 0.99 for stage I, II, III, and IV, respectively (Figure 7f), further confirming the robust performance of PME for early cancer diagnostics. The ability to quantify methylation levels in human serum underscores the potential of PME for cancer diagnosis, treatment monitoring, and disease progression tracking, offering a non‐invasive and highly sensitive approach for personalized healthcare. Furthermore, its adaptability to biological samples highlights its clinical relevance for detecting circulating tumor DNA and other disease‐related epigenetic modifications, positioning PME as a promising diagnostic tool for future clinical applications.

## 3 Conclusion

We developed a highly sensitive and adaptable PME‐assisted SERS method as facile strategy for trapping and label‐free detection of DNA, enabling the recognition of epigenetic modifications such as DNA methylation. This approach generates hotspots regions around target analytes, effectively addressing mismatched geometrical properties and producing a strong electromagnetic field that results in significant SERS signal enhancement. The quantification of DNA methylation was further explored through SERS intensity analysis across various methylation levels and ratios, demonstrating the reliability of this technique for DNA analysis. Additionally, the feasibility of this method was validated with clinical serum samples of colorectal cancer and normal patients. A LR‐based machine learning model successfully quantified and classified different methylation levels in human serum with high sensitivity, specificity, and accuracy, demonstrating the capability of this method for global methylation quantification in biofluids. These results highlight the potential of the PME method as a reliable and versatile technique for precise label‐free sensing, with broad applicability to diverse target analytes in both clinical and analytical fields.

## 4 Experimental Section

### Materials

Hydroxylamine (HA), sodium citrate dihydrate, HH, gold chloride hydrate (HAuCl_4_), ethanol (EtOH), 4‐aminothiophenol (4‐ATP), and human serum were purchased from Sigma‐Aldrich (St. Louis, MO, USA). Synthetic single strand DNA were purchased from Bioneer (Daejeon, South Korea). Diethylpyrocarbonate (DEPC) water was purchased from Tech & Innovation (Gangwon, South Korea). Polystyrene 96‐well‐plate in 85.40 × 127.60 × 14.40 mm dimension (well‐dimension: 6.92 × 10.80 mm) was purchased from SPL Life Sciences Co., LTD (Gyeonggi, South Korea).

### Fabrication AuS

To fabricate Au nanosponge (AuS), the chemical reduction method in the previous paper was applied.^[^ ^37^ ^]^ First, AuS was deposited on the polystyrene substrate of 96‐well plate through a chemical reaction mixture of HAuCl_4_ precursor and HA solutions, which were prepared in the EtOH solvent at a final concentration ratio of 1:10. The mixture solution with a volume of 200 µL was added to each well and left until the supernatant was completely dried, followed by a washing process with EtOH and distilled (DI) water.

### Material Characterization

Surface morphology images were characterized by FE‐SEM (JSM‐6700, JEOL) and the cross‐section images and grain structures were characterized by HR‐TEM (JEM‐2200FS with Image Cs‐corrector, JEOL). The protrusion size distribution plots were obtained by measuring the size of protrusion in SEM images using ImageJ software (NIH Image, Bathesda, MD). XRD spectrum was obtained for AuS using XRD instrument (Panalaytical X'Pert Pro). UV–vis absorbance spectra were measured via UV–vis spectroscopy (SpectraMax M2).

### SERS Activity Test

The SERS performance of PME was evaluated using AuS coated 96‐well plate platform. A 100 µL of 4‐ATP solution (10^−7^ m) was added to AuS substrate, followed by addition of 50 µL of HH (80 mm) and 50 µL of HAuCl_4_ (40 mm). The reaction was allowed to proceed for 2 min until the solution became completely clear and transparent. The samples were then directly measured by Raman spectroscopy without any further treatment. To determine the LOD of PME, 100 µL of 4‐ATP solutions at various concentrations (ranging from 10^−7^ to 10^−13^ m) were added to the wells, following the same procedure. The LOD values were calculated using the formula LOD = 3σ S^−1^, where σ represents the standard deviation of blank measurement (*n* = 5) at the analytic peak, and S represents the slope of the standard curve. To determine the EF of PME, we measured 100 µL of 20 mm 4‐ATP solution on a bare substrate and compared it with a 10 nm 4‐ATP measurement using PME. The EF value was calculated using the formula of EF = (I_SERS_/I_Bare_)(N_Bare_/N_SERS_), where I_SERS_ and I_Bare_ are the Raman intensity of 4‐ATP from PME and bare substrate, respectively, and N_SERS_ and N_Bare_ are the average number of molecules producing the I_SERS_ and I_Bare_, respectively. Real‐time monitoring of molecular behavior was performed by treating 4‐ATP 10^−7^ m with the specified protocols and observing it with Raman spectroscopy for 500 s. All Raman measurements were conducted using an Ocean Optics portable probe spectrometer system (UQEPRO‐Raman) with laser wavelength of 785 nm, laser powers of 40 mW, and exposure time of 10 s.

### Simulation of Electromagnetic Field Distribution

The E‐field distribution was theoretically calculated using the FDTD Solution software tool (version 8.21, Lumerical Solutions). The geometric parameters were constructed based on the magnified SEM and TEM images where the morphologies and sizes of AuS structures are defined for modeling. The polarized incident light (785 nm) was vertically shined on the surface. The periodic boundary conditions were applied to the x and y axes, the metal boundary was applied to the positive z axis. The mesh scale was fixed at 0.25 nm. The dielectric constant of Au was −21.6448 + *i*0.743321 at 785 nm, and the refractive index of air (background) was set to 1. The real‐time E‐field generation was recorded as videos with and without DNA.

### SERS Measurement of DNA

To investigate the capability of PME for DNA analysis, single‐stranded DNA (ssDNA) with sequence of 5’‐GCAAAAGCAAGCTGAACCCGAA‐3 were diluted in DEPC water. A 100 µL of ssDNA sample (100 ng mL⁻^1^) was added to AuS platform, followed by addition of 50 µL of HH (500 mm) and 50 µL of HAuCl_4_ (50 mm). The reaction was left for 10 min until the solution became completely clear and transparent. The samples were then directly measured by Raman spectroscopy without any further treatment. To determine the LOD of PME, 100 µL of ssDNA sample at various concentrations (ranging from 500 fg mL⁻^1^ to 100 ng mL⁻^1^) was added to the wells, following the same procedure. The uniformity and reproducibility of the Raman signal was assessed by measuring Raman signal of ssDNA (100 µL of 100 ng mL⁻^1^) at 100 different points and across 10 different AuS substrates, respectively, after 10 min of reaction time. The established protocols were also implemented for DNA methylation profiling. All Raman measurement were conducted with a laser wavelength of 785 nm, laser powers of 40 mW, and exposure time of 60 s.

### SERS Measurement for Quantification DNA Methylation

To investigate the capability of PME for DNA methylation analysis, methylated ssDNA with sequence of 5’‐G[5‐methyl dC]AAAAG[5‐methyl dC]AAGC[5‐methyl dC]TGAAC[5‐methyl dC]CGAA‐3 were diluted in DEPC water. A 100 µL of methylated ssDNA sample (100 ng mL⁻^1^) was added to AuS platform, followed by addition of 50 µL of HH (500 mm) and 50 µL of HAuCl_4_ (50 mm). The reaction was left for 10 min until the solution became completely clear and transparent. The samples were then directly measured by Raman spectroscopy without any further treatment. To quantify the methylation levels of DNA methylation, A 100 µL of methylated ssDNA with methylation degree ranging from 0 to 4 (50 ng mL⁻^1^) were added to the wells, following the same procedure. The methylation ratios of DNA methylation were also quantified with the same procedure, utilizing various DNA length ranging from 22 to 42 nt (50 ng mL⁻^1^) with the same methylation level of 4.

All Raman measurement were conducted with a laser wavelength of 785 nm, laser powers of 40 mW, and exposure time of 60 s.

### SERS Measurement of DNA Methylation in Human Serum

To investigate the feasibility of PME, unmethylated and methylated ssDNA derived from a colon cancer of Homo sapiens BRCA1 gene with sequence of 5’‐GCAAAAGCAAGCTGAACCCGAA‐3 were spiked into 100 µL of human serum with final concentration of 10 ng mL⁻^1^. A 100 µL of spiked ssDNA samples were added to AuS platform, followed by addition of 50 µL of HH (500 mm) and 50 µL HAuCl_4_ (50 mm). The reaction was left for 10 min until the solution became completely clear and transparent. The samples were then directly measured by Raman spectroscopy without any further treatment. The same protocols were also implemented for methylation level analysis in human serum. All Raman measurements were conducted with a laser wavelength of 785 nm, laser powers of 40 mW, and exposure time of 60 s.

### SERS Measurement of Clinical Samples

To investigate the practical application of PME, clinical serum samples of colorectal cancer and normal patients from Samsung Medical Center were measured without any pre‐treatment. A 100 µL of serum samples were added to AuS platform, followed by addition of 50 µL of HH (500 mm) and 50 µL HAuCl_4_ (50 mm). The reaction was left for 10 min until the solution became completely clear and transparent. The samples were then directly measured by Raman spectroscopy without any further treatment. The same protocols were also implemented for methylation level analysis in human serum. All Raman measurements were conducted with a laser wavelength of 785 nm, laser powers of 40 mW, and exposure time of 60 s.

### Machine Learning Analysis

A supervised machine learning model was developed using the open‐source Phyton package scikit‐learn (sklearn), applying LR for precise and efficient category differentiation. To determine the most relevant Raman shifts, the complete Raman spectrum (600–1 700 cm^−1^) was used as input features. A two‐step feature selection approach was implemented: first statistical testing (*t*‐test, *p* < 0.05) was applied to identify significant spectral differences, followed by multivariate analysis using partial least square discrimination analysis (PLS‐DA) with a VIP threshold > 1.4 to refine the feature selection. For DNA methylation quantification, only the most critical Raman peaks within 1 420–1 500 cm^−1^ were segmented and used as input features. After feature selection, the dataset was split into 70% training and 30% testing to ensure robust model evaluation. Raman intensities were converted into methylation levels using a mathematical regression model. The LR model was implemented to predict methylation levels by applying logistic function to a linear combination of input features. The LR mechanism involves calculating a weighted sum of the input features, adding a bias term, and applying the sigmoid function to map this sum to probability between 0 and 1. Model performance was evaluated using the MAE and R^2^ score to assess the accuracy of predicted and methylation ratios relative to actual values. The trained LR model was then used to predict methylation levels in clinical samples by applying the calibrated methylation ratio dataset as a reference.

### Statistical Analysis

Each sample was measured ten times. The collected Raman spectra were normalized to the ν(PO_2_^−^) peak at 1 048 cm^−1^. Statistical analyses were performed using Python. All results are presented as mean ± standard deviation (SD). An independent samples *t*‐test was used to assess differences between two groups, while one‐way analysis of variance (ANOVA) followed by Tukey's post‐hoc test was applied for multiple group comparisons. A *p*‐value of ≤ 0.05 was considered statistically significant.

### Ethics Approval

Clinical serum samples from colorectal cancer patients and normal controls were obtained from Samsung Medical Center, with approval from the Institutional Review Board (IRB approval number: SMC 2021‐06‐083‐015). Informed written consent was obtained from all human participants prior to the study.

## Conflict of Interest

The authors declare no conflict of interest.

## Supporting information



Supporting Information

Supplemental Video 1

Supplemental Video 2

## Data Availability

The data that support the findings of this study are available from the corresponding author upon reasonable request.
