# Supplementary material for: Plasmonic Molecular Entrapment for Label‐Free Methylated DNA Detection and Machine‐Learning Assisted Quantification
Source: Adv Sci (Weinh). 2025 May 8;12(29):2503257. doi: 10.1002/advs.202503257 (PMC12362744; doi:10.1002/advs.202503257)
Supplement: Supplementary file 1 — Supporting Information [file ADVS-12-2503257-s001.docx]

Supporting Information

Plasmonic molecular entrapment for label-free methylated DNA detection and machine-learning assisted quantification

Muhammad Shalahuddin Al Ja’farawy, Vo Thi Nhat Linh, Chaewon Mun, Jun-Yeong Yang, Jun Young Kim, Rowoon Park, Sung-Gyu Park, Dong-Ho Kim, Min-Young Lee^*^, and Ho Sang Jung^*^

M.S.A. Ja’farawy, Dr. V.T.N. Linh, C. Mun, Dr. J.-Y. Yang, J.-Y. Kim, Dr. R. Park, Dr. S.-G. Park, Dr. D.-H. Kim, Dr. M.-Y. Lee, Dr. H. S. Jung

Advanced Bio and Healthcare Materials Research Division, Korea Institute of Materials Science (KIMS), Changwon, Gyeongnam 51508, South Korea

E-mail: myay0615@kims.re.kr (Dr. Min-Young Lee) and jhs0626@kims.re.kr (Dr. Ho Sang Jung)

M.S.A. Ja’farawy, Prof. D.-H. Kim, Prof. H. S. Jung

Advanced Materials Engineering, Korea National University of Science and Technology (UST), Daejeon, 34113, South Korea

Prof. H. S. Jung

School of Convergence Science and Technology, Medical Science and Engineering, Pohang University of Science and Technology (POSTECH), Pohang, Kyungbuk 37673, South Korea


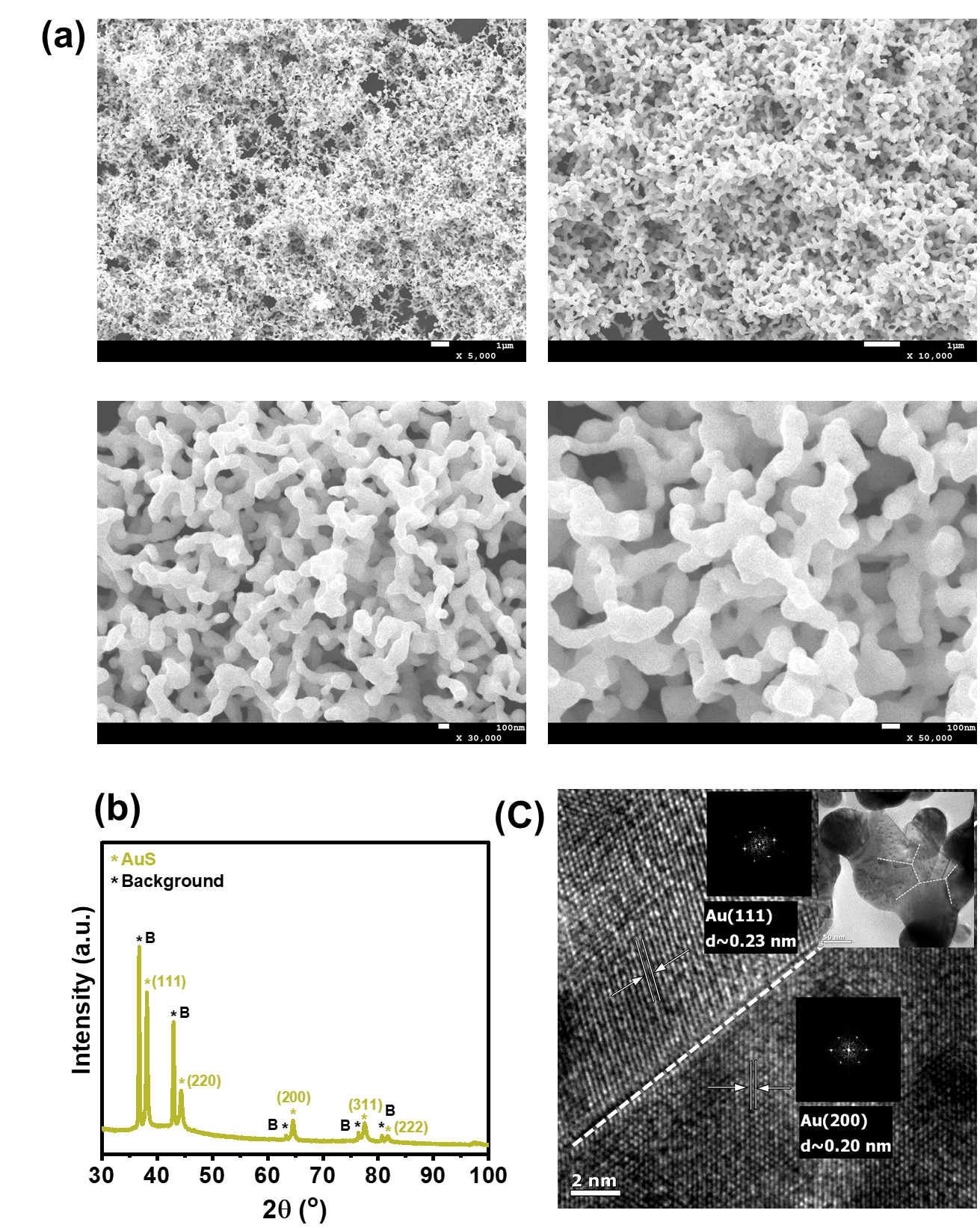


**Figure S1.** Material characterization of pristine AuS. (a) SEM image in various magnification, (b) XRD measurement, and (c) TEM analysis of AuS.


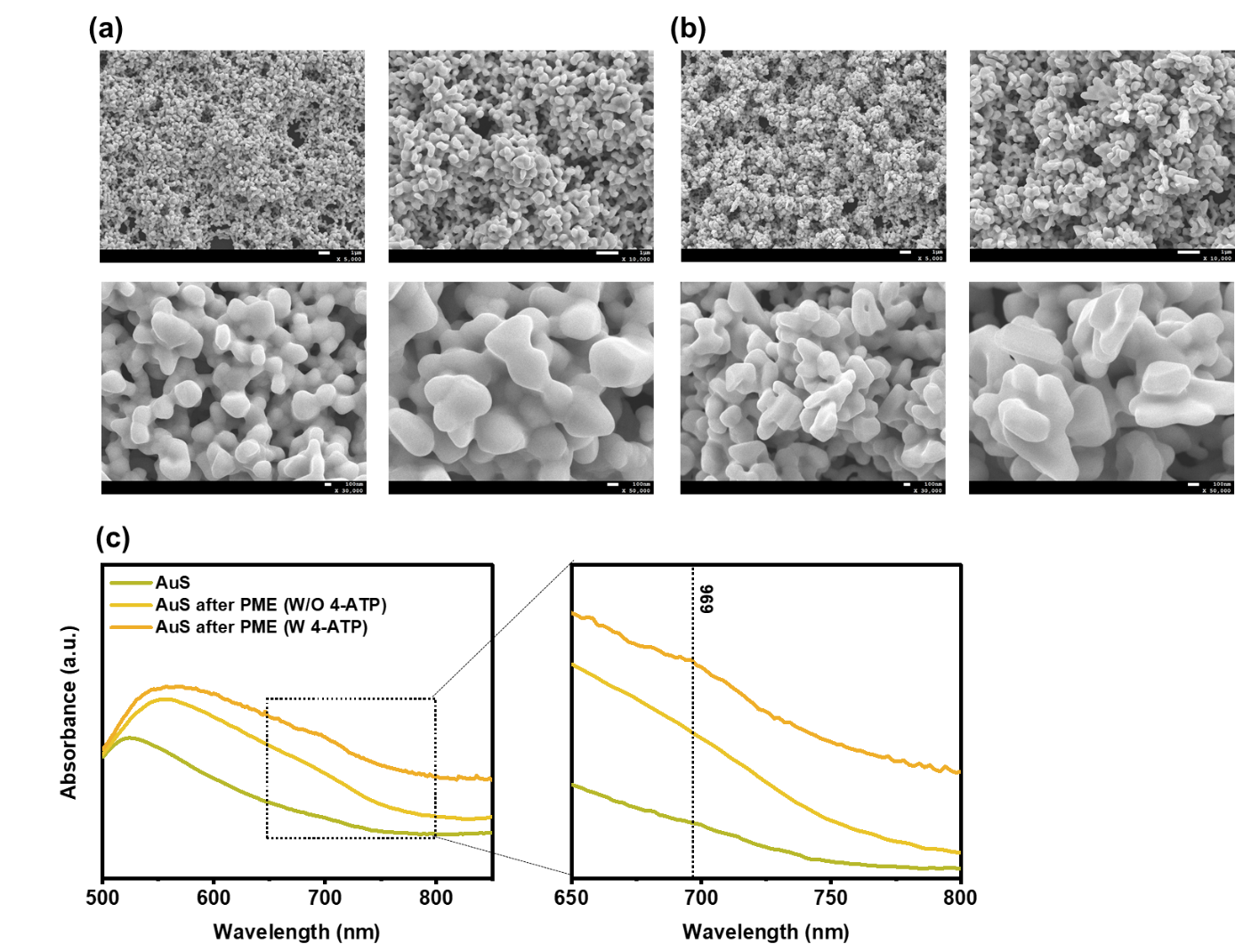


**Figure S2.** Material analysis of AuS after PME. SEM image of AuS after PME in various magnification (a) without target analytes, (b) with target analytes, and (c) UV-vis measurement of AuS at before and after PME in both absence and presence of target analytes.


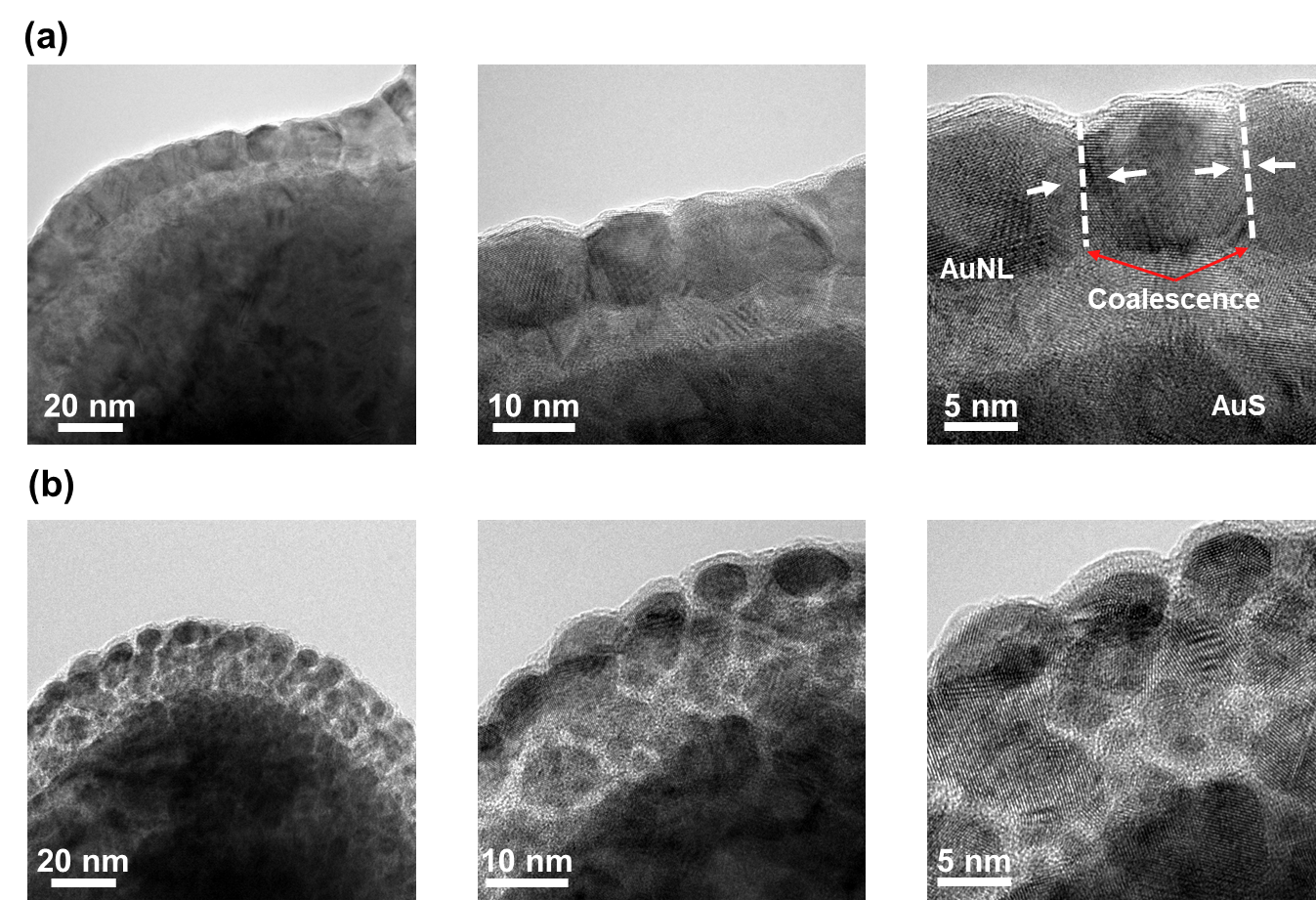


**Figure S3.** TEM analysis of the laminated area on AuS surface (a) without target analytes and (b) with target analytes.


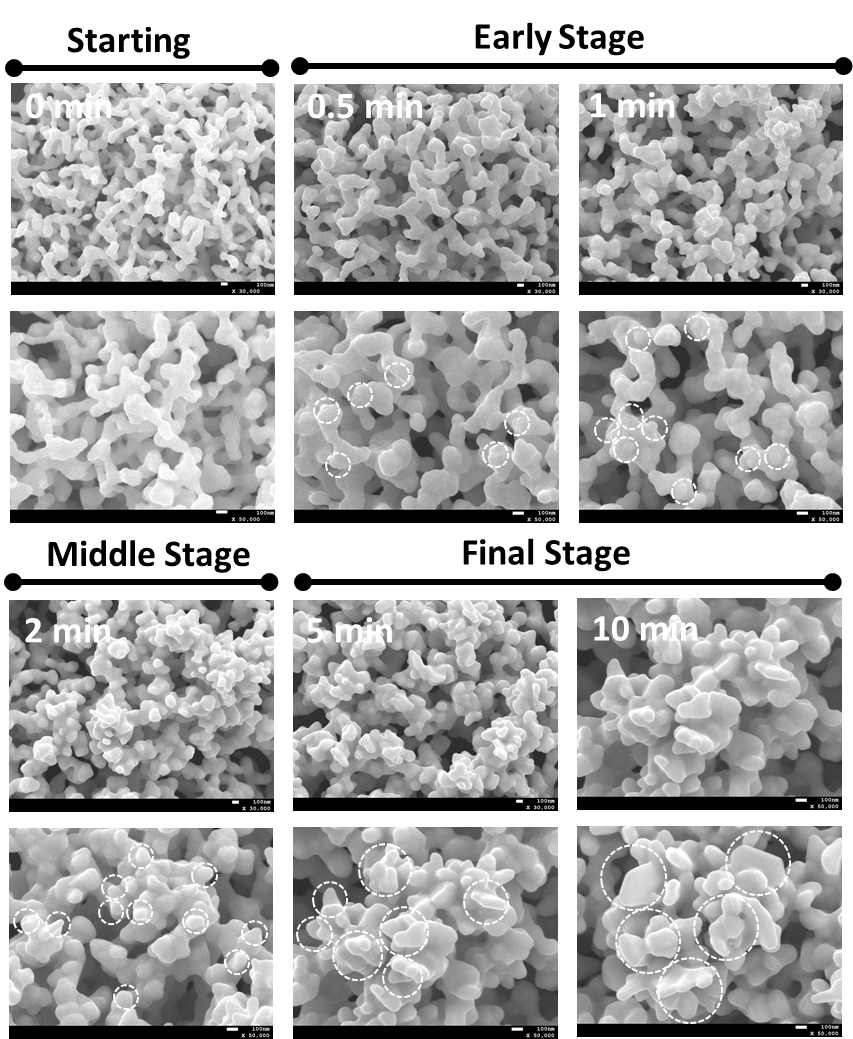


**Figure S4.** SEM images of AuS at different PME reaction time (protrusion growths are marked with white marker).


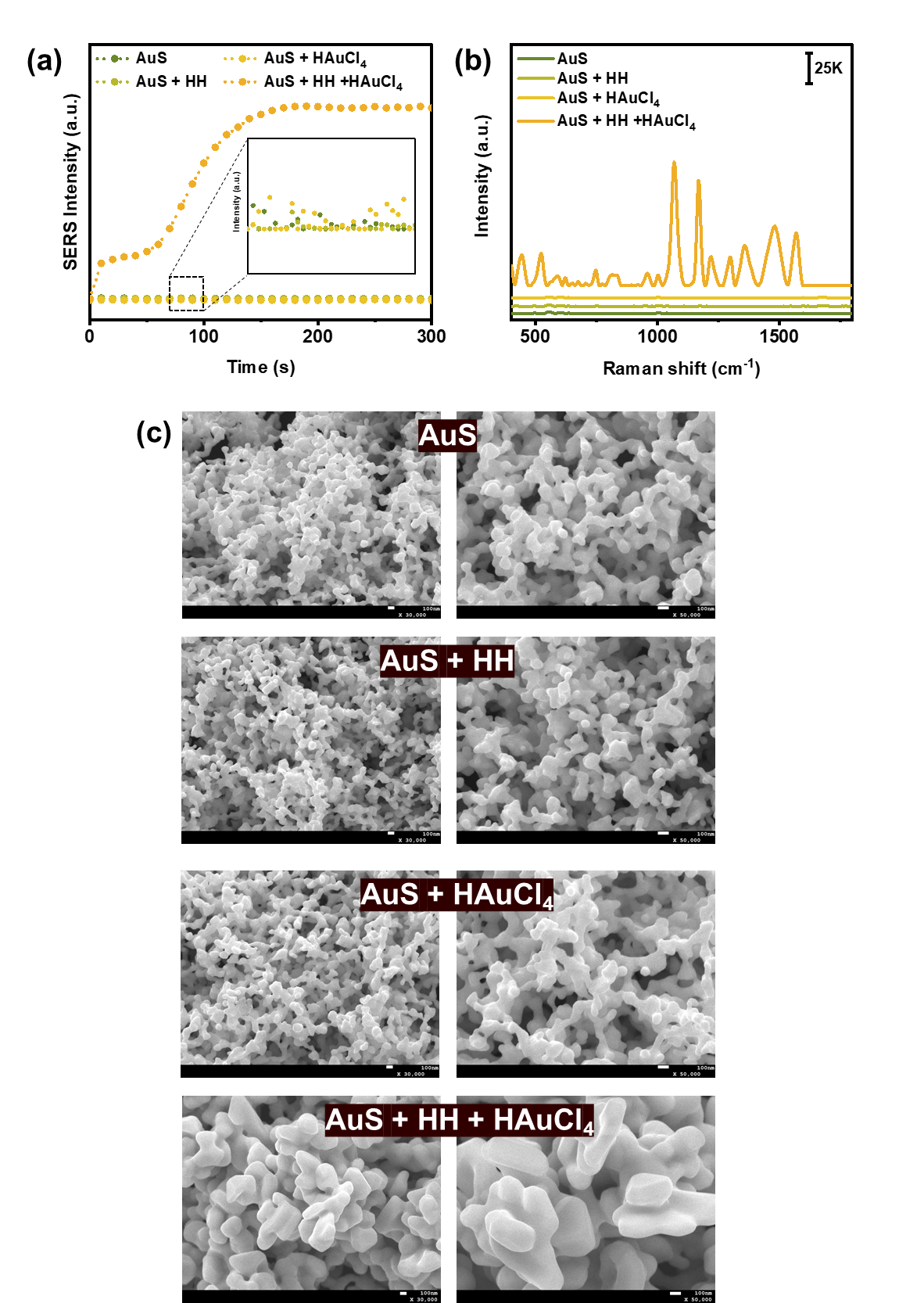


**Figure S5.** (a) Real-time SERS signal monitoring at 1071 cm^-1^, (b) SERS spectra of 4-ATP (scale bar refers to SERS intensity), and (c) SEM images recorded at t = 300 s under different conditions: AuS, AuS+HH, AuS+HAuCl_4_, and AuS+HH+HAuCl_4_.


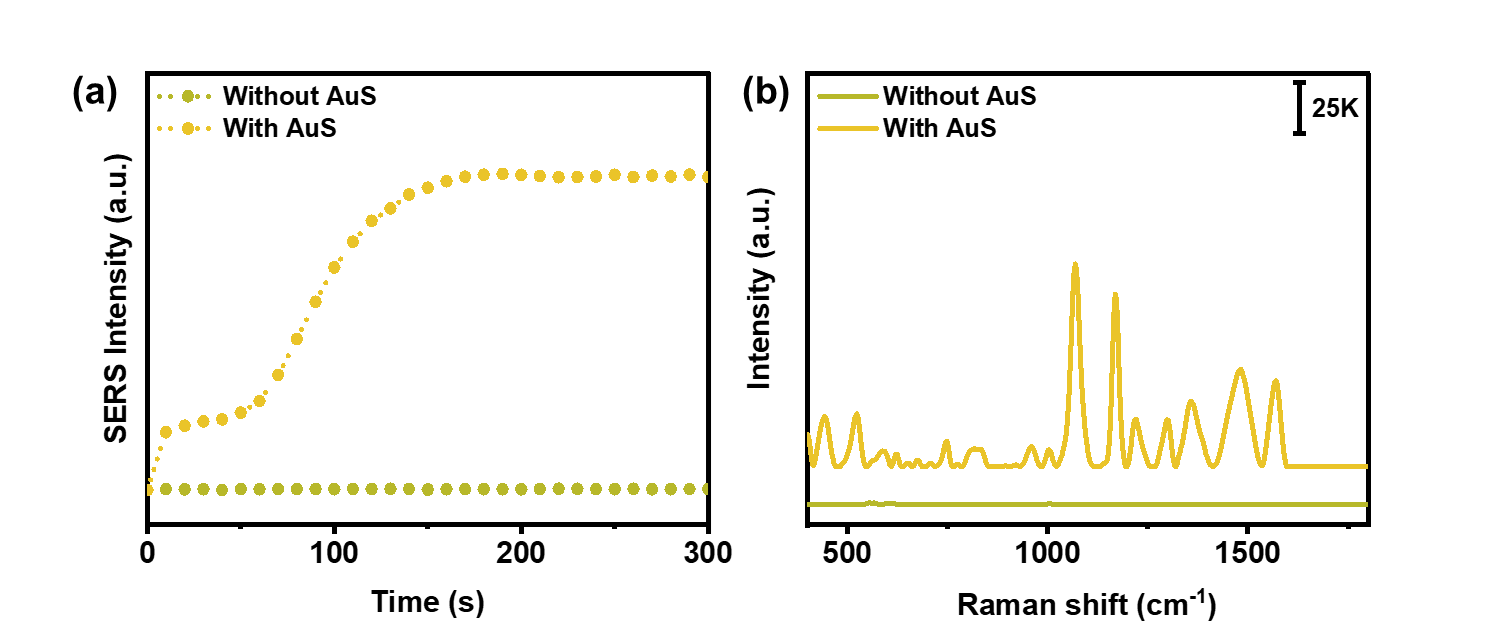


**Figure S6.** (a) Real-time SERS signal monitoring at 1071 cm^-1^ and (b) SERS spectra of 4-ATP recorded at t = 300 s under different conditions: PME method in the presence and absence of nucleation site AuS (scale bar refers to SERS intensity).


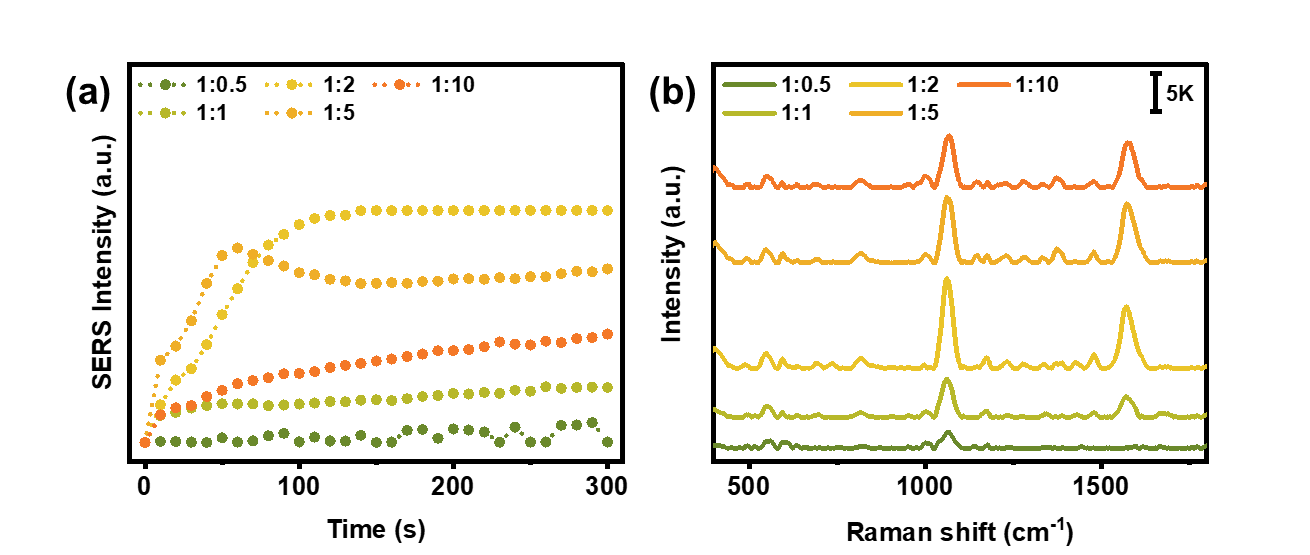


**Figure S7.** (a) Real-time SERS signal monitoring at 1071 cm^-1^ and (b) SERS spectra of 4-ATP recorded at t = 300 s under different conditions: PME method in various trapping agent HAuCl_4_:HH ratios (scale bar refers to SERS intensity).


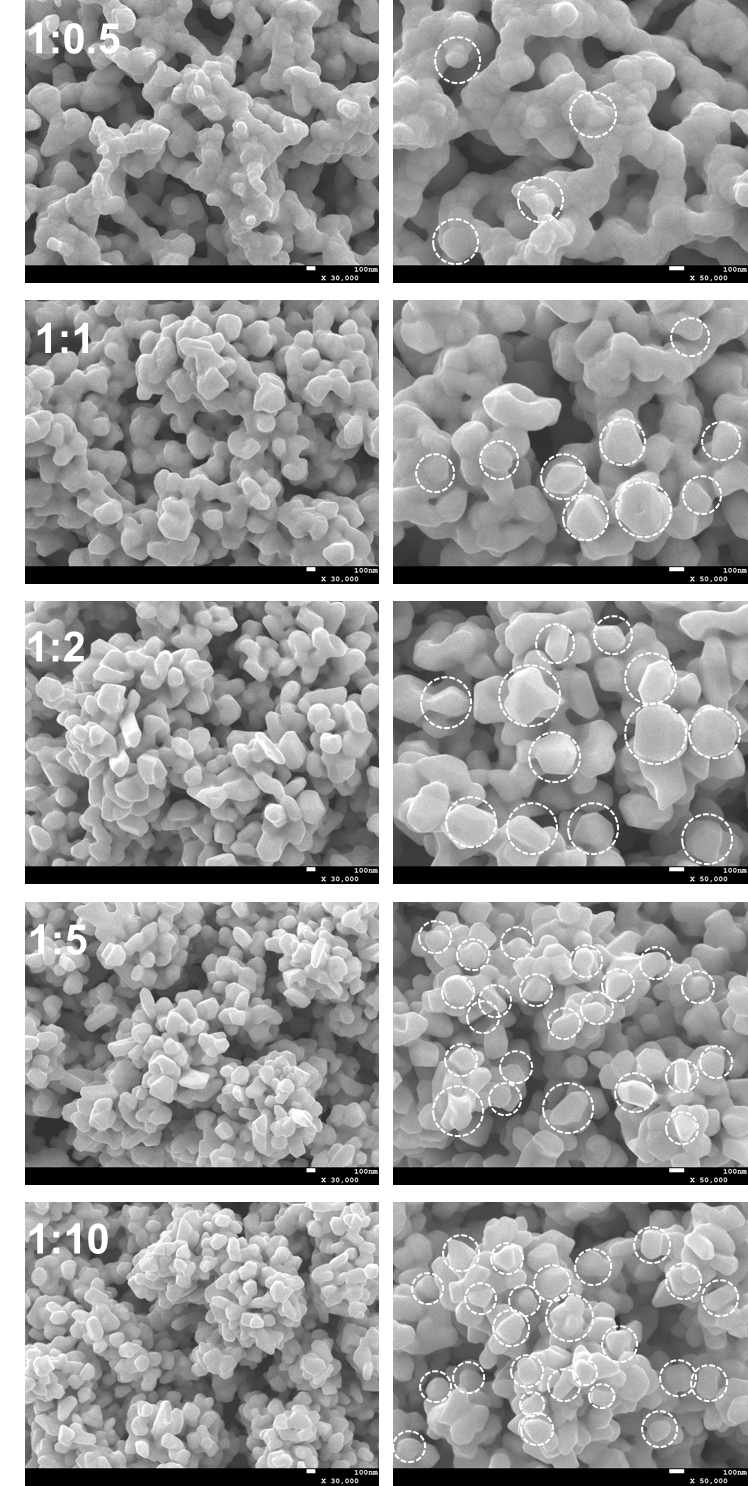


**Figure S8.** SEM images of AuS after PME reaction for 300 s in various HAuCl_4_:HH ratios.


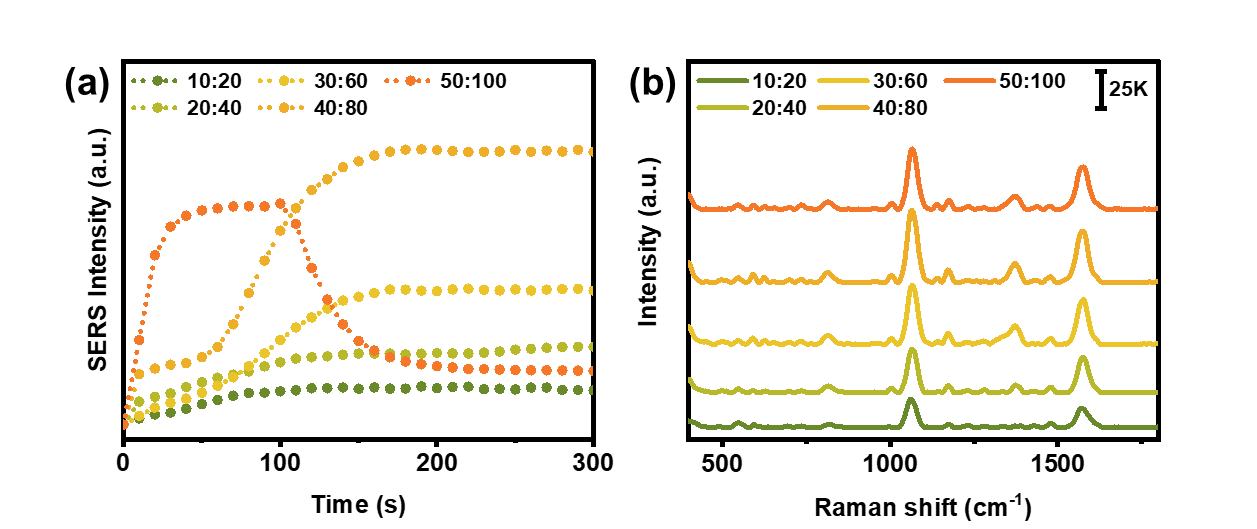


**Figure S9.** (a) Real-time SERS signal monitoring at 1071 cm^-1^ and (b) SERS spectra of 4-ATP recorded at t = 300 s under different conditions: PME method in various HAuCl_4_:HH concentrations (in mM) at same ratio of 1:2 (scale bar refers to SERS intensity).


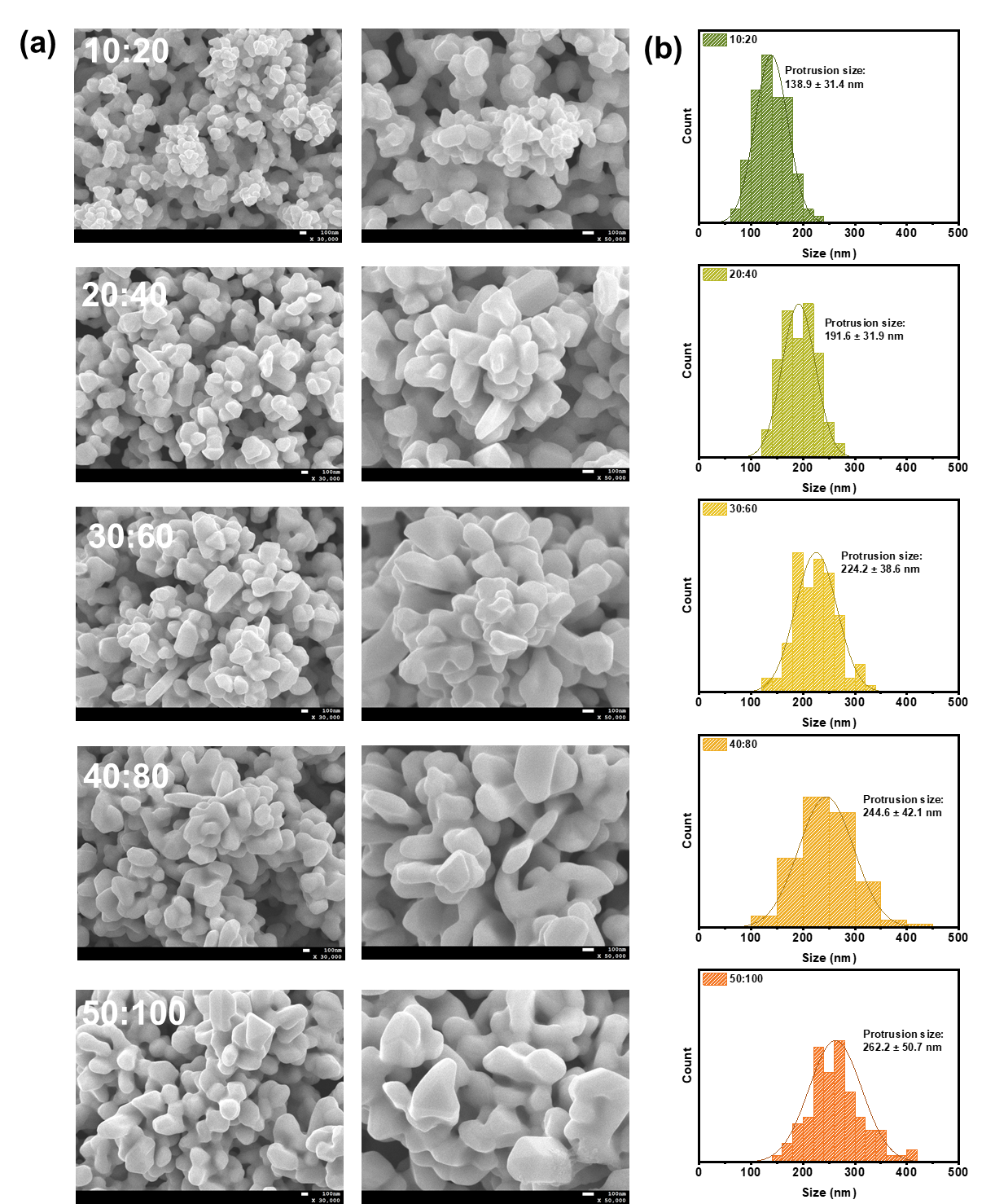


**Figure S10.** (a) SEM images of AuS after PME reaction for 300 s and (b) protrusion size distribution in various HAuCl_4_:HH concentration.


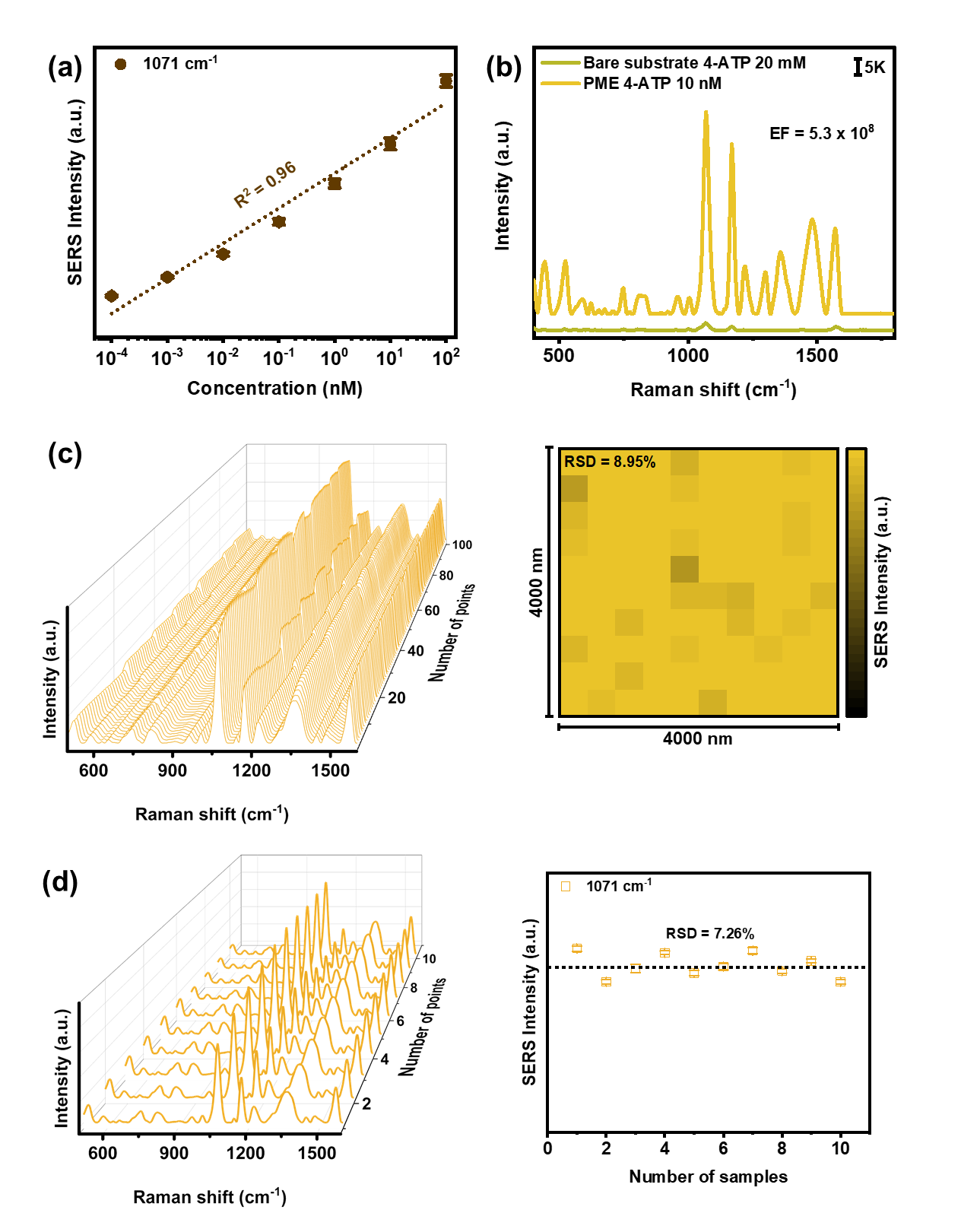


**Figure S11.** SERS performance of PME. (a) standard curve, (b) enhancement factor (scale bar refers to SERS intensity), (c) signal uniformity, and (d) reproducibility test of 4-ATP in PME method


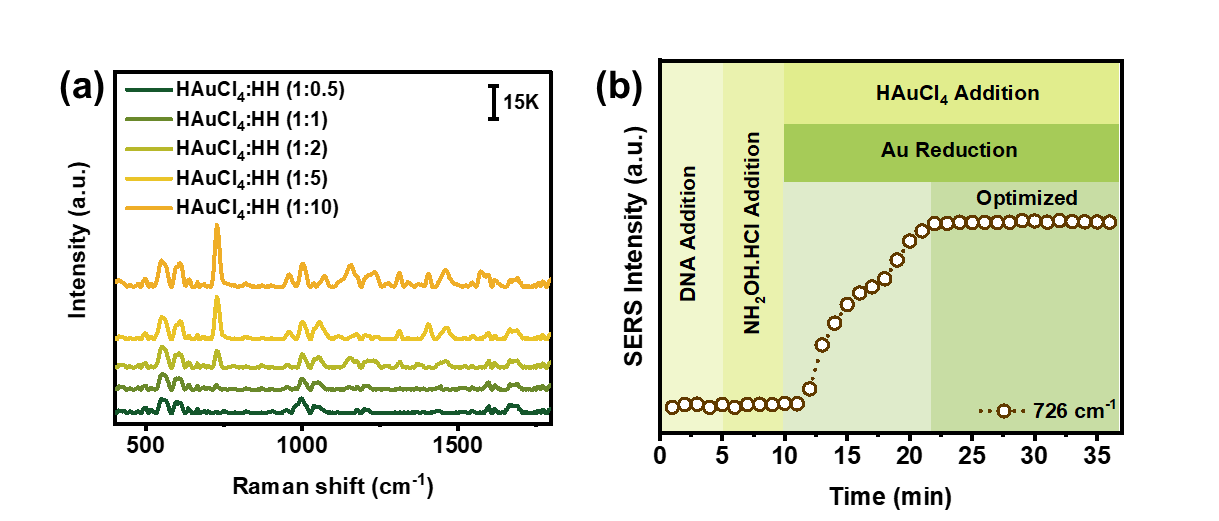


**Figure S12.** Optimization PME method condition for DNA detection. (a) SERS spectra comparison of DNA in various HAuCl_4_:HH ratios (scale bar refers to SERS intensity) and (b) Real-time SERS signal monitoring at 726 cm^-1^.


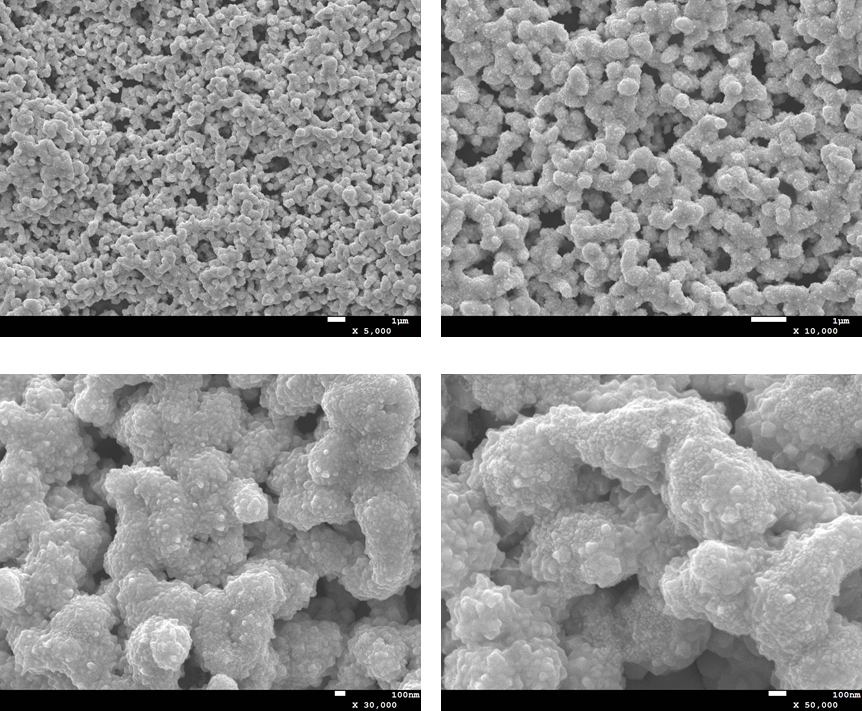


**Figure S13.** SEM image of AuS after PME in the presence of DNA with various magnification.


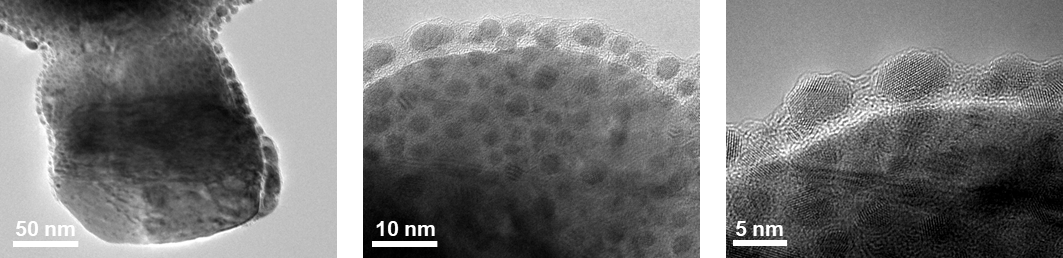


**Figure S14.** TEM images of AuS after PME in the presence of DNA with various magnification.


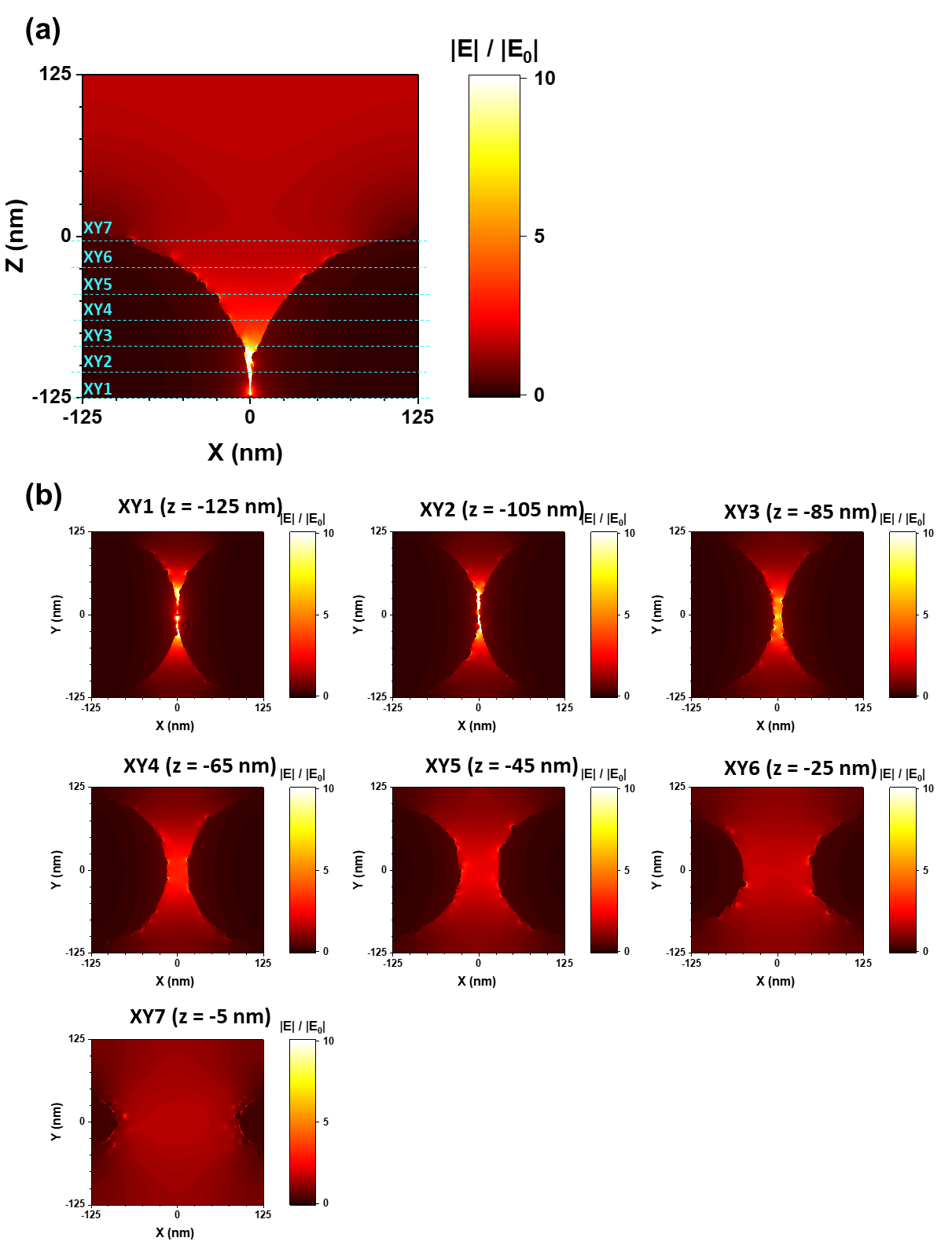


**Figure S15.** E-field distribution of AuS surface after PME (without DNA) obtained by FDTD simulation at (a) XZ cross-section view and (b) in various z axis position.


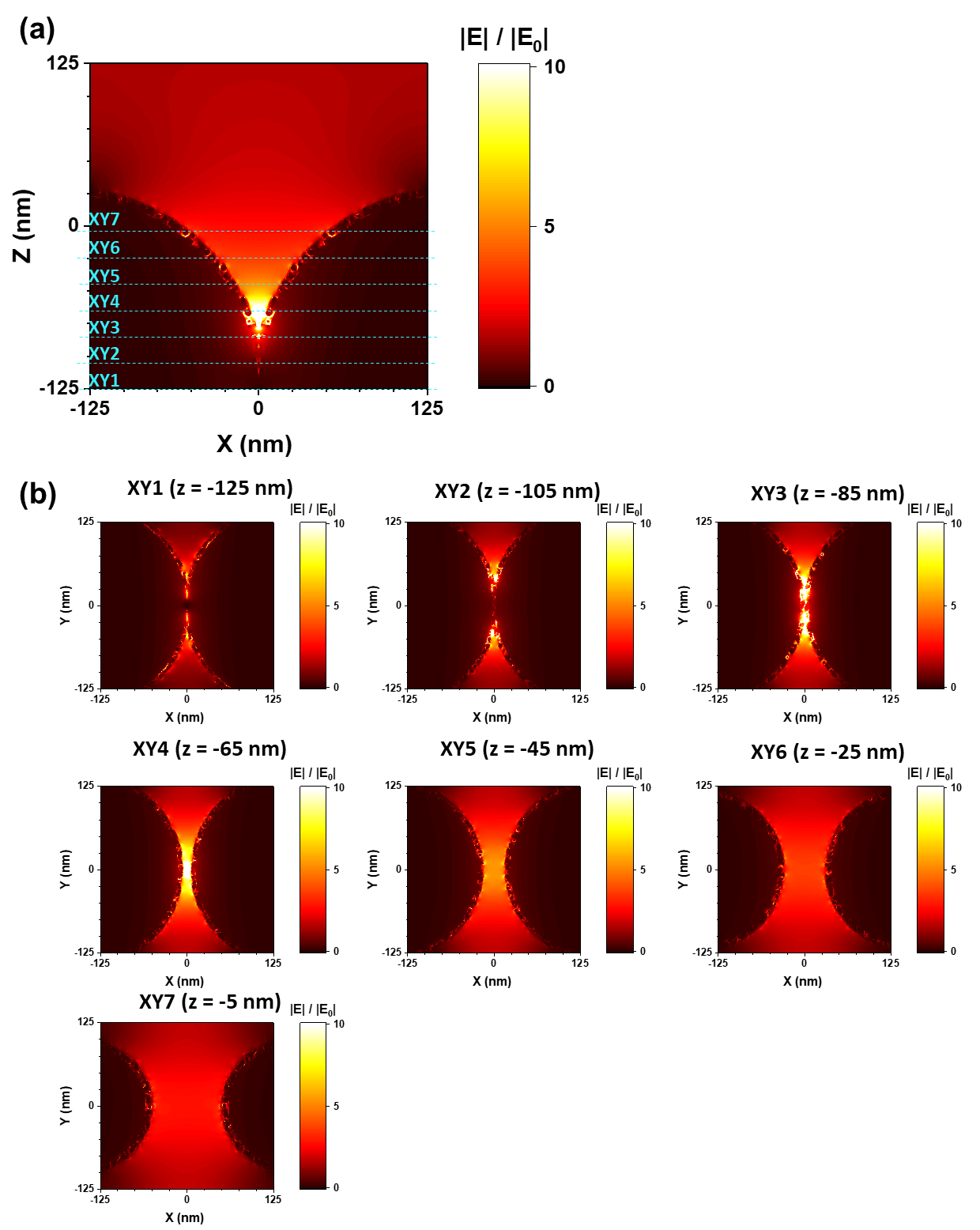


**Figure S16.** E-field distribution of AuS surface after PME (with DNA) obtained by FDTD simulation at (a) XZ cross-section view and (b) in various z axis position.


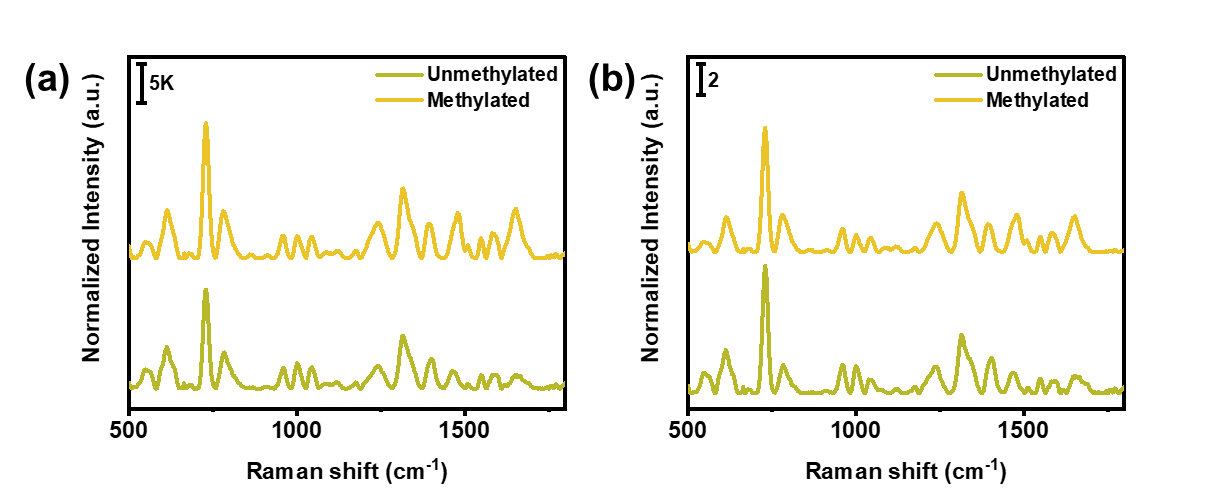


**Figure S17.** SERS spectra of unmethylated and methylated DNA (a) before (scale bar refers to SERS intensity) and (b) after normalization (scale bar refers to normalized SERS intensity).

**
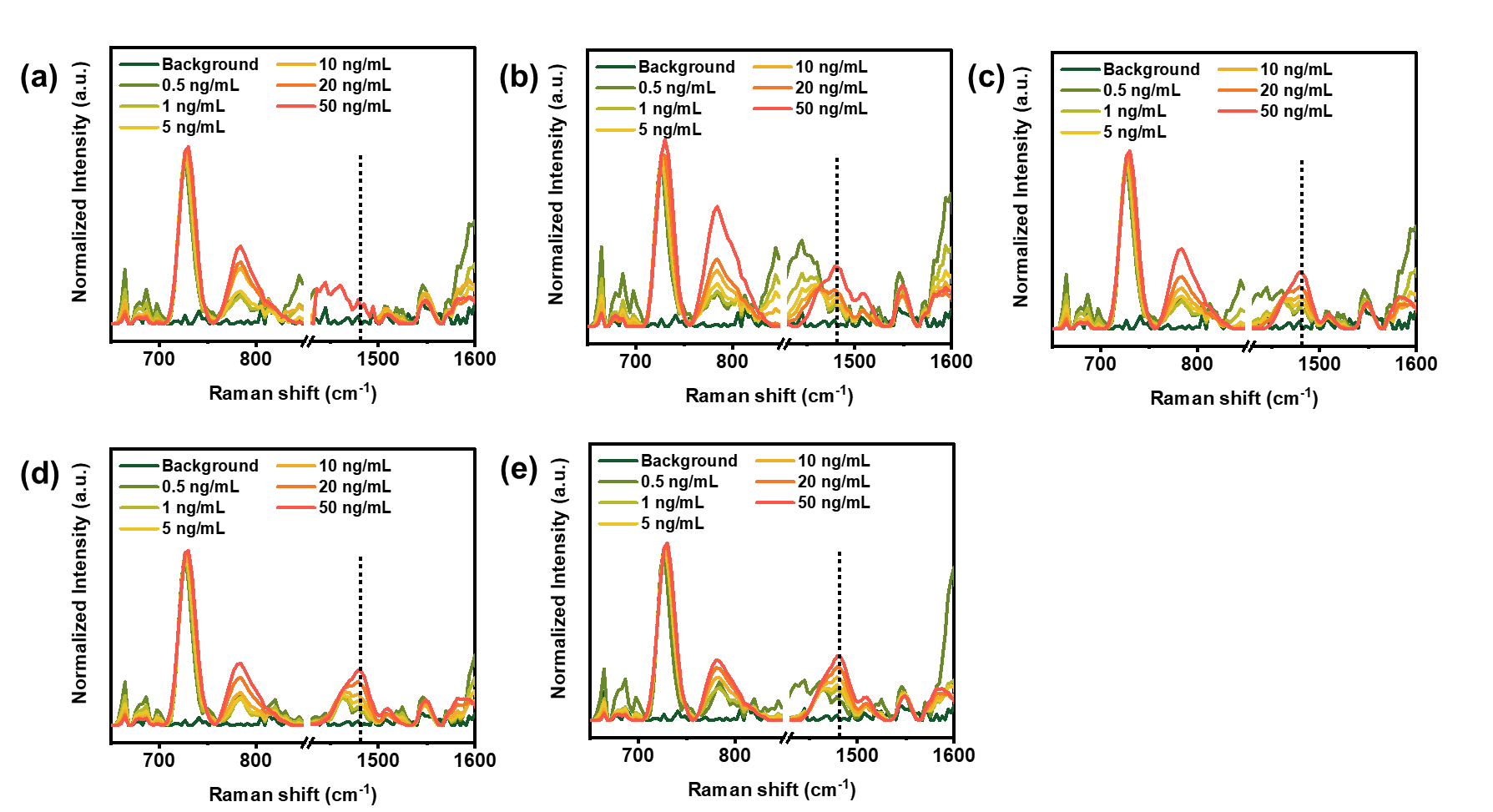
**

**Figure S18.** SERS spectra of (a) 0-methylation, (b) 1-methylation, (c) 2-methylation, (d) 3-methylation, and (e) 4-methylation in various concentration.


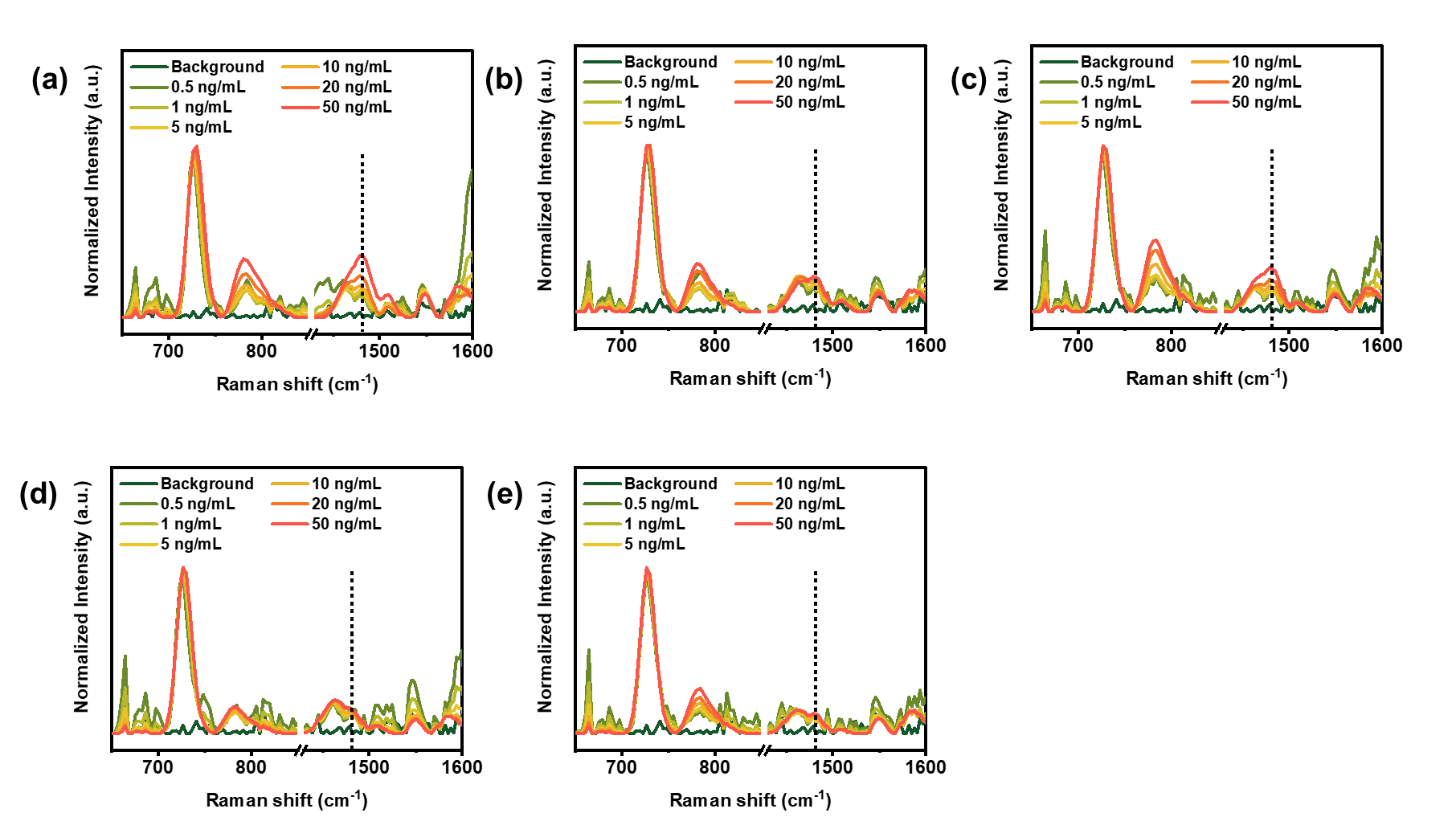


**Figure S19.** SERS spectra of various DNA methylation ratios (a) 18.2%, (b) 14.8%, (c) 12.5%, (d) 10.8%, and (e) 9.5% in various concentration.


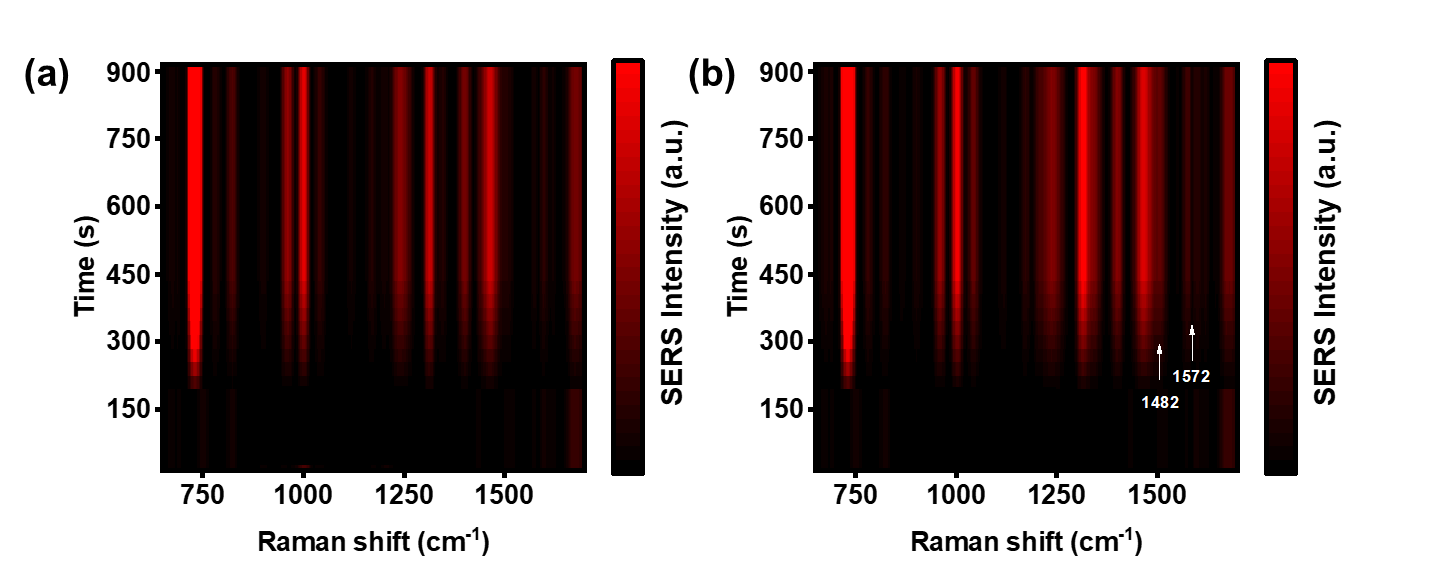


**Figure S20.** Temporal evolution of real-time monitoring of SERS spectra from (a) unmethylated and (b) methylated DNA in human serum.


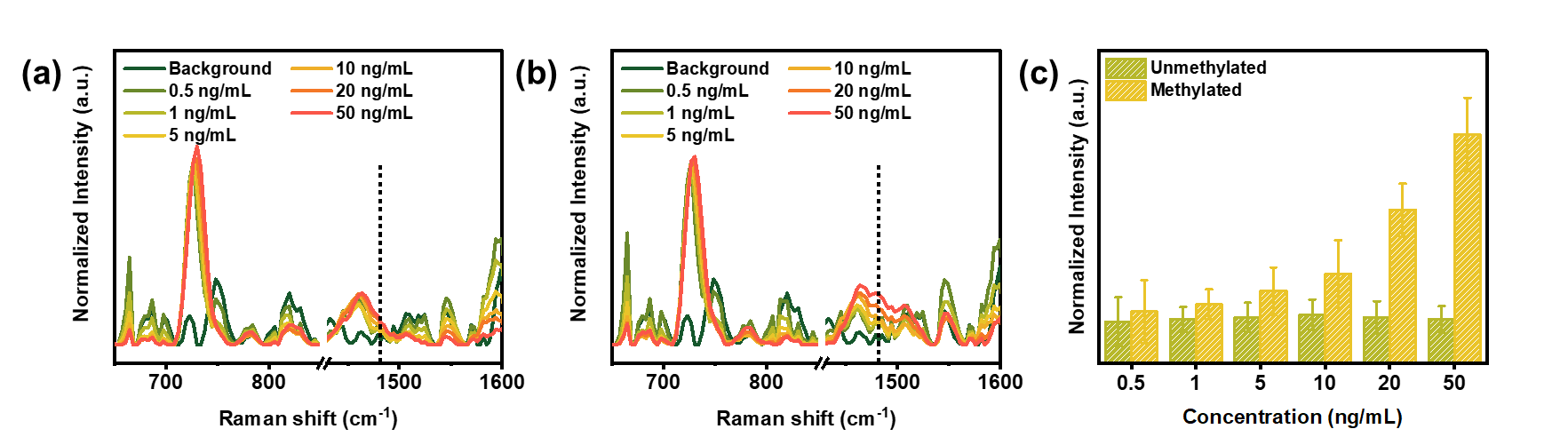


**Figure S21.** SERS spectra of of (a) unmethylated, (b) methylated DNA in human serum in various concentration, and (c) its Raman intensity at 1482 cm^-1^ (error bar refers to standard deviation).

**
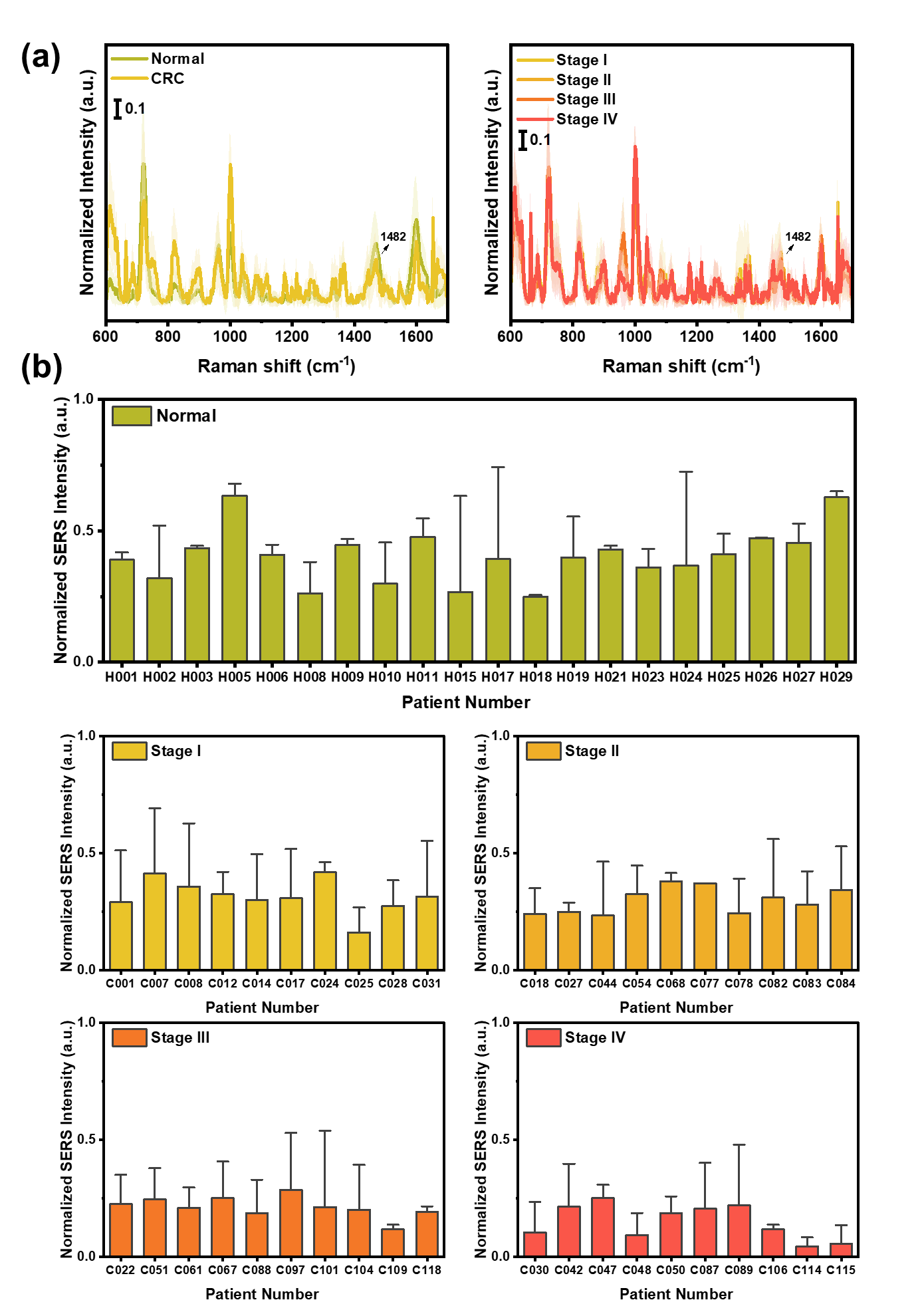
**

**Figure S22.** (a) SERS spectra (scale bar refers to normalized SERS intensity) and (b) SERS intensity comparison at 1482 cm^-1^ of clinical serum from normal and CRC patients (error bar refers to standard deviation).\


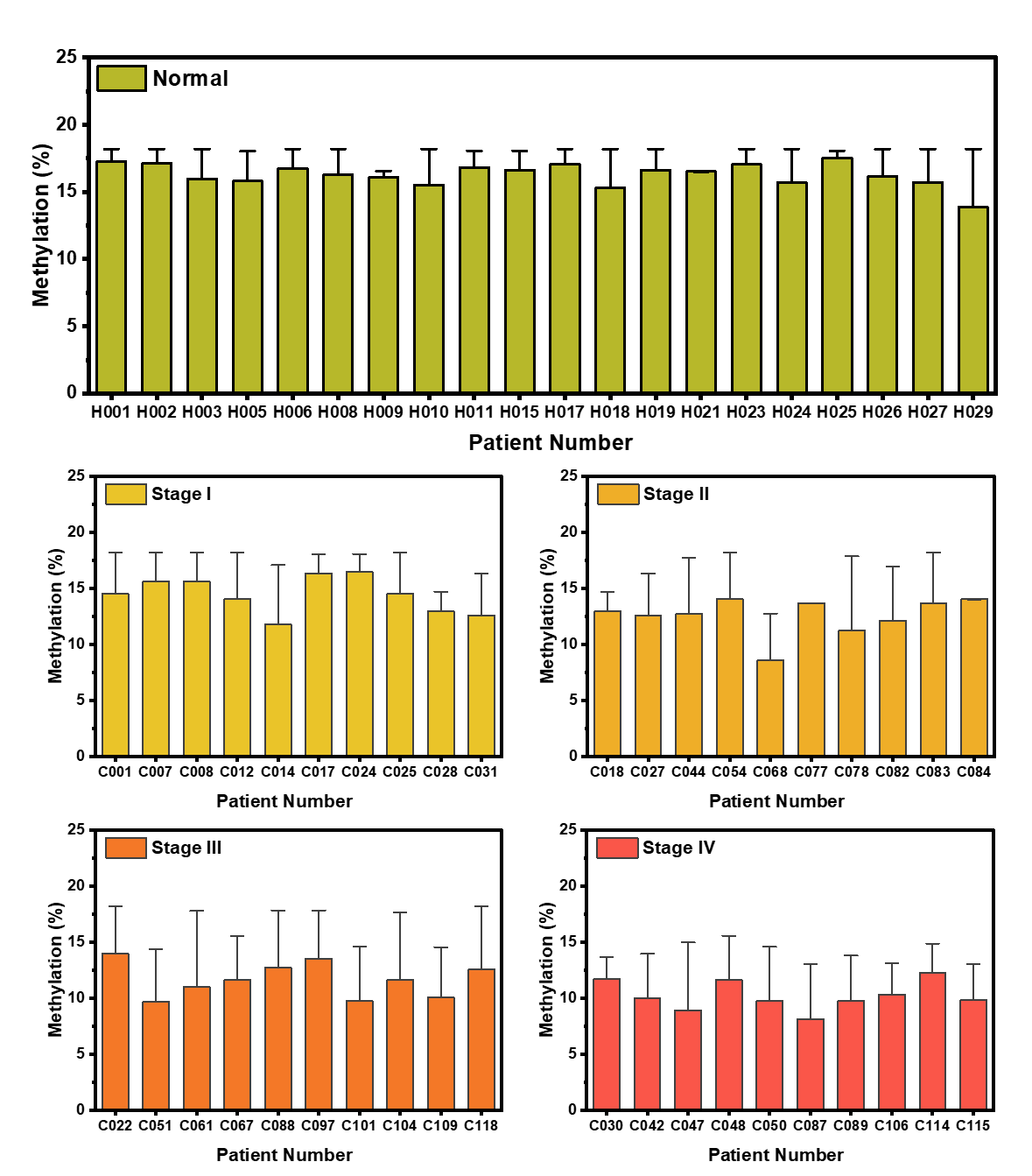


**Figure S23.** Global DNA methylation quantification in clinical serum samples from normal and CRC patients using PME-assisted machine learning (error bar refers to standard deviation).


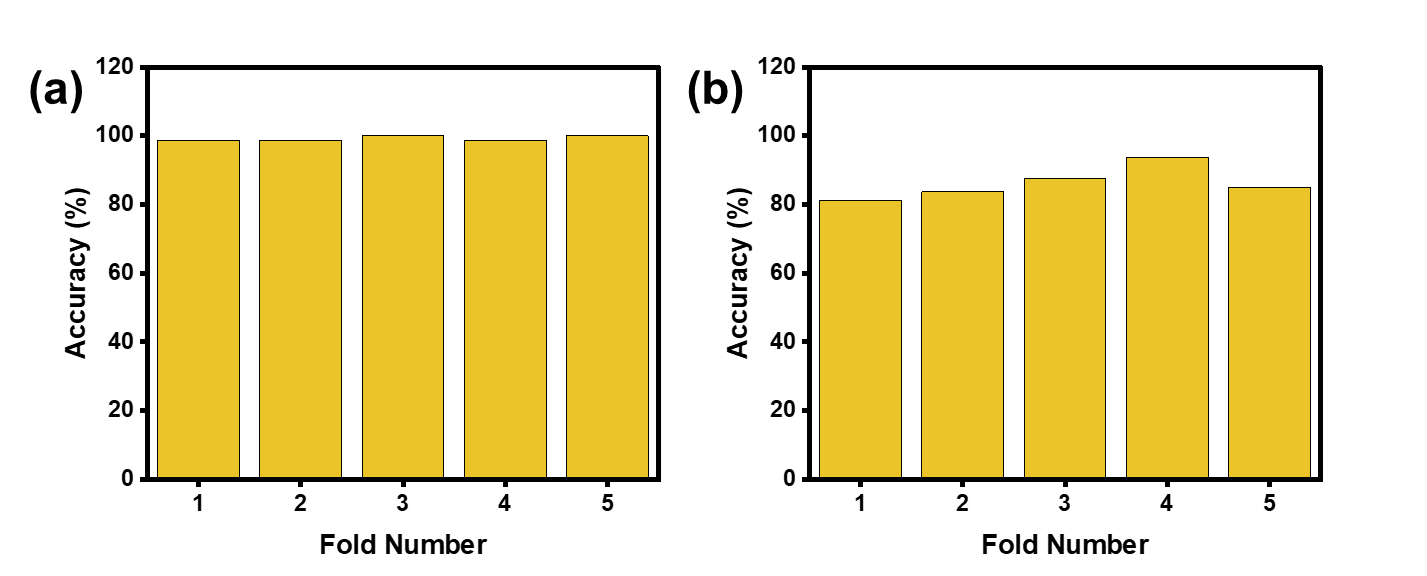


**Figure S24.** 5-fold cross-validation of the LR model for classifying (a) normal versus CRC and (b) among different CRC stages.

**Table S1.** Performance comparison of conventional diagnostic tools and the developed method in this work for DNA methylation.

| **Sensing Technique** | **Accuracy** | **Sensitivity** | **Cost (USD/sample)** | **Time** | **Reference** |
| --- | --- | --- | --- | --- | --- |
| Whole genome bisulfite sequencing (WGBS) | ~90-95% | ~95-99% | $500-1500 | 48-72 h | ^[1-2]^ |
| Reduced representation bisulfite sequencing (RRBS) | ~90% | ~95% | $100-500 | 24-48 h | ^[2-3]^ |
| Enzymatic methyl sequencing (EM-Seq) | ~90-95% | ~95% | $300-1000 | 24-48 h | ^[2, 4-5]^ |
| Oxford nanopore sequencing | ~90-95% | ~95% | $100-300 | 6-12 h | ^[6-7]^ |
| Surface-enahanced Raman spectroscopy | ~85-90% | ~90-95% | - | 1-2 h | ^[8-9]^ |
| Fluorescence-based assay | ~85-90% | ~90-95% | $50-200 | 2-6 h | ^[8, 10]^ |
| Electrochemical biosensor | ~80-90% | ~90% | - | 1-2 h | ^[11-12]^ |
| **This work** | 99.2% | 100% | < $ 1 | few minutes |  |

**Table S2.** Raman peak assignment of 4-ATP before and after PME

| **Raman shift (cm^-1^)** | | **Peak Assignment** | **Ref.** |
| --- | --- | --- | --- |
| **Before PME** | **After PME** |  |  |
| - | 442 | ν(C-N) + ν(C-S) + γ(CCC) | ^[13-14]^ |
| - | 1003 | γ(CC) + γ(CCC) | ^[13-14]^ |
| 1071 | 1071 | ν(C-S) | ^[13-14]^ |
| 1171 | 1171 | δ(C-H) | ^[13-14]^ |
| - | 1217 | ν(C-N) | ^[13]^ |
| - | 1301 | ν(C-N) | ^[13]^ |
| - | 1358 | ν(CC) + δ(CH) | ^[14]^ |
| - | 1482 | γ(C-C), δ(C-S) | ^[13-14]^ |
| 1569 | 1569 | γ(C-C) | ^[13-14]^ |

**Table S3.** Raman peak assignment of DNA before and after PME

| **Raman shift (cm^-1^)** | | **Peak Assignment** | **Ref.** |
| --- | --- | --- | --- |
| **Before PME** | **After PME** |  |  |
| 612 | 612 | Ring breathing – G-dRib | ^[15]^ |
| 726 | 726 | Ring breathing - A | ^[15]^ |
| - | 782 | Ring breathing - C | ^[15]^ |
| - | 963 | dRib | ^[16]^ |
| 1000 | 1000 | T, G, C, dRib | ^[15]^ |
| 1048 | 1048 | ν(PO_2_^-^) | ^[16]^ |
| - | 1233 | Ring mode - T | ^[15-16]^ |
|  | 1313 | Ring mode - A, C | ^[15-16]^ |
| - | 1404 | δ(2’CH_2_) - dRib | ^[15]^ |
| - | 1460 | Ring mode - C | ^[16]^ |
| - | 1548 | ν(C4C3), ν(C5C4) – G, A, C | ^[15]^ |
| - | 1593 | Ring mode – A, G | ^[16]^ |
| - | 1652 | ν(C4=O) – T, G, C | ^[15]^ |

*dRib:deoxyribose sugar, A: adenine, G: guanine, C: cytosine, T: thymine.*

**Table S4.** Sequences of the DNA in various size

| **No** | **Sequence** | **Size (nt)** |
| --- | --- | --- |
| 1 | 5’-GCAAAAGCAAGCTGAACCCGAA-3 | 22 |
| 2 | 5'-GCAAAAGCAAGCTGAACCCGAAGCTTC-3 | 27 |
| 3 | 5'-GCTTCGCAAAAGCAAGCTGAACCCGAAGCTTC-3 | 32 |
| 4 | 5'-AAGCTTCGCAAAAGCAAGCTGAACCCGAAGCTTCAAA-3 | 37 |
| 5 | 5’-TCGAAGCTTCGCAAAAGCAAGCTGAACCCGAAGCTTCAAACG-3 | 42 |

**Table S5.** Sequences of the DNA in various methylation number

| **No** | **Sequence** | **Number of Methylation** |
| --- | --- | --- |
| 1 | 5’-GCAAAAGCAAGCTGAACCCGAA-3 | 0 |
| 2 | 5’-G[5-methyl dC]AAAAGCAAGCTGAACCCGAA-3 | 1 |
| 3 | 5’-G[5-methyl dC]AAAAG[5-methyl dC]AAGCTGAACCCGAA-3 | 2 |
| 4 | 5’-G[5-methyl dC]AAAAG[5-methyl dC]AAGC[5-methyl dC]TGAACCCGAA-3 | 3 |
| 5 | 5’-G[5-methyl dC]AAAAG[5-methyl dC]AAGC[5-methyl dC]TGAAC[5-methyl dC]CGAA-3 | 4 |

**Table S6.** Sequences of the methylated DNA in various size

| **No** | **Sequence** | **Size (nt)** | **Methylation Ratio (%)** |
| --- | --- | --- | --- |
| 1 | 5’-G[5-methyl dC]AAAAG[5-methyl dC]AAGC[5-methyl dC]TGAAC[5-methyl dC]CGAA-3 | 22 | 18.2 |
| 2 | 5’-G[5-methyl dC]AAAAG[5-methyl dC]AAGC[5-methyl dC]TGAAC[5-methyl dC]CGAAGCTTC-3 | 27 | 14.8 |
| 3 | 5’-GCTTCG[5-methyl dC]AAAAG[5-methyl dC]AAGC[5-methyl dC]TGAAC[5-methyl dC]CGAAGCTTC-3 | 32 | 12.5 |
| 4 | 5’-AAGCTTCG[5-methyl dC]AAAAG[5-methyl dC]AAGC[5-methyl dC]TGAAC[5-methyl dC]CGAAGCTTCAAA-3 | 37 | 10.8 |
| 5 | 5’-TCGAAGCTTCG[5-methyl dC]AAAAG[5-methyl dC]AAGC[5-methyl dC]TGAAC[5-methyl dC]CGAAGCTTCAAA-CG-3 | 42 | 9.5 |

**Table S7.** Clinical characteristics of the patients involved in this study

| **Classification** | **Patient** | **Stage** | **CEA** | **Patient** | **Stage** | **CEA** |
| --- | --- | --- | --- | --- | --- | --- |
| **Colorectal Cancer** | C001 | Stage I | NA | C017 | Stage I | 1.53 |
|  | C007 |  | 2.68 | C024 |  | 2.72 |
|  | C008 |  | 4.07 | C025 |  | NA |
|  | C012 |  | 2.35 | C028 |  | 2.66 |
|  | C014 |  | 3.69 | C031 |  | 1.51 |
|  | C018 | Stage II | 2.45 | C077 | Stage II | 4.32 |
|  | C027 |  | 3.94 | C078 |  | 3.71 |
|  | C044 |  | 3.35 | C082 |  | 4.13 |
|  | C054 |  | 0.8 | C083 |  | 1.37 |
|  | C068 |  | 5.07 | C084 |  | 3.12 |
|  | C022 | Stage III | 2.55 | C097 | Stage III | 1.47 |
|  | C051 |  | 2.11 | C101 |  | 2.34 |
|  | C061 |  | 3.72 | C104 |  | 0.88 |
|  | C067 |  | 1.44 | C109 |  | 1.41 |
|  | C088 |  | 5.07 | C118 |  | 10.3 |
|  | C030 | Stage IV | 80.6 | C087 | Stage IV | 1382 |
|  | C042 |  | 187 | C089 |  | 8.59 |
|  | C047 |  | 0.68 | C106 |  | 133 |
|  | C048 |  | 6.43 | C114 |  | 0.77 |
|  | C050 |  | 5.64 | C115 |  | 35.7 |
| **Normal** | H001 |  | NA | H017 |  | NA |
|  | H002 |  | NA | H018 |  | NA |
|  | H003 |  | NA | H019 |  | NA |
|  | H005 |  | NA | H021 |  | NA |
|  | H006 |  | NA | H023 |  | NA |
|  | H008 |  | NA | H024 |  | NA |
|  | H009 |  | NA | H025 |  | NA |
|  | H010 |  | NA | H026 |  | NA |
|  | H011 |  | NA | H027 |  | NA |
|  | H015 |  | NA | H029 |  | NA |

**References List**

[1] W. Zhang, T. D. Spector, P. Deloukas, J. T. Bell, B. E. Engelhardt, Predicting genome-wide DNA methylation using methylation marks, genomic position, and DNA regulatory elements, *Genome Biol.* **2015**, 16, 14.

[2] R. Sun, P. Zhu, Advances in measuring DNA methylation, *Blood Sci.* **2022**, 4, 8.

[3] Y. Liu, Y. Han, L. Zhou, X. Pan, X. Sun, Y. Liu, M. Liang, J. Qin, Y. Lu, P. Liu, A comprehensive evaluation of computational tools to identify differential methylation regions using RRBS data, *Genomics* **2020**, 112, 4567.

[4] D. R. Masser, A. S. Berg, W. M. Freeman, Focused, high accuracy 5-methylcytosine quantitation with base resolution by benchtop next-generation sequencing, *Epigenetics Chromatin* **2013**, 6, 33.

[5] Š. Šestáková, C. Šálek, H. Remešová, DNA Methylation Validation Methods: a Coherent Review with Practical Comparison, *Biol. Proced. Online* **2019**, 21, 19.

[6] Y. Liu, W. Rosikiewicz, Z. Pan, N. Jillette, P. Wang, A. Taghbalout, J. Foox, C. Mason, M. Carroll, A. Cheng, S. Li, DNA methylation-calling tools for Oxford Nanopore sequencing: a survey and human epigenome-wide evaluation, *Genome Biol.* **2021**, 22, 295.

[7] Q. Gouil, A. Keniry, Latest techniques to study DNA methylation, *Essays Biochem.* **2019**, 63, 639.

[8] M. Nazmul Islam, S. Yadav, M. Hakimul Haque, A. Munaz, F. Islam, M. S. Al Hossain, V. Gopalan, A. K. Lam, N.-T. Nguyen, M. J. A. Shiddiky, Optical biosensing strategies for DNA methylation analysis, *Biosens. Bioelectron.* **2017**, 92, 668.

[9] P. Zheng, P. Raj, L. Liang, L. Wu, S. K. Paidi, J. H. Kim, I. Barman, Label-free plasmonic spectral profiling of serum DNA, *Biosens. Bioelectron.* **2024**, 254, 116199.

[10] H. Jiang, X. Yang, M. Mi, X. Wei, H. Wu, Y. Xin, L. Jiao, S. Sun, C. Sun, Development and performance evaluation of TaqMan real-time fluorescence quantitative methylation specific PCR for detecting methylation level of PER2, *Mol. Biol. Rep.* **2022**, 49, 2097.

[11] J. Huang, S. Zhang, F. Mo, S. Su, X. Chen, Y. Li, L. Fang, H. Huang, J. Deng, H. Liu, X. Yang, J. Zheng, An electrochemical DNA biosensor analytic technique for identifying DNA methylation specific sites and quantify DNA methylation level, *Biosens. Bioelectron.* **2019**, 127, 155.

[12] F. Chen, X. Wang, X. Cao, Y. Zhao, Accurate Electrochemistry Analysis of Circulating Methylated DNA from Clinical Plasma Based on Paired-End Tagging and Amplifications, *Anal. Chem.* **2017**, 89, 10468.

[13] N. R. Tiwari, M. Y. Liu, S. Kulkarni, Y. Fang, Study of adsorption behavior of aminothiophenols on gold nanorods using surface-enhanced Raman spectroscopy, *J. Nanophotonics* **2011**, 5, 053513.

[14] X. Hu, T. Wang, L. Wang, S. Dong, Surface-Enhanced Raman Scattering of 4-Aminothiophenol Self-Assembled Monolayers in Sandwich Structure with Nanoparticle Shape Dependence:  Off-Surface Plasmon Resonance Condition, *J. Phys. Chem. C* **2007**, 111, 6962.

[15] G. K. Chandra, C. Eklouh-Molinier, M. Fere, J.-F. Angiboust, C. Gobinet, L. Van-Gulick, P. Jeannesson, O. Piot, Probing in Vitro Ribose Induced DNA-Glycation Using Raman Microspectroscopy, *Anal. Chem.* **2015**, 87, 2655.

[16] A. J. Hobro, M. Rouhi, E. W. Blanch, G. L. Conn, Raman and Raman optical activity (ROA) analysis of RNA structural motifs in Domain I of the EMCV IRES, *Nucleic Acids Res.* **2007**, 35, 1169.
